# Numerical Study of Atrial Fibrillation Effects on Flow Distribution in Aortic Circulation

**DOI:** 10.1007/s10439-020-02448-6

**Published:** 2020-01-14

**Authors:** Amin Deyranlou, Josephine H. Naish, Christopher A. Miller, Alistair Revell, Amir Keshmiri

**Affiliations:** 1 Department of Mechanical, Aerospace and Civil Engineering (MACE), The University of Manchester, Manchester, M13 9PL UK; 2 Division of Cardiovascular Sciences, School of Medical Sciences, Faculty of Biology, Medicine and Health, The University of Manchester, Manchester, M13 9PL UK; 3 Division of Cardiovascular Sciences, School of Medical Sciences, Faculty of Biology, Medicine and Health, University of Manchester, Manchester Academic Health Science Centre, Oxford Road, Manchester, M13 9PL UK; 4 Manchester University NHS Foundation Trust, Manchester Academic Health Science Centre, Southmoor Road, Wythenshawe, Manchester, M13 9PL UK; 5 Wellcome Centre for Cell-Matrix Research, Division of Cell-Matrix Biology & Regenerative Medicine, School of Biology, Faculty of Biology, Medicine and Health, University of Manchester, Manchester Academic Health Science Centre, Oxford Road, Manchester, M13 9PL UK

**Keywords:** Atrial fibrillation, Aorta, 4D phase contrast magnetic resonance imaging, Computational fluid dynamics

## Abstract

Atrial fibrillation (AF) is the most common type of arrhythmia, which undermines cardiac function. Atrial fibrillation is a multi-facet malady and it may occur as a result of other diseases or it may trigger other problems. One of the main complications of AF is stroke due to the possibility of clot formation inside the atrium. However, the possibility of stroke occurrence due to the AF and the location from which an embolus dispatches are subject of debate. Another hypothesis about the embolus formation during AF is thrombus formation in aorta and carotid arteries, embolus detachment and its movement. To investigate the possibility of the latter postulation, the current work suggests a parametric study to quantify the sensitivity of aortic flow to four common AF traits including lack of atrial kick, atrial remodelling, left ventricle systolic dysfunction, and high frequency fibrillation. The simulation was carried out by coupling several in-house codes and ANSYS-CFX module. The results reveal that AF traits lower flow rate at left ventricular outflow tract, which in general lowers blood perfusion to systemic, cerebral and coronary circulations. Consequently, it leads to endothelial cell activation potential (ECAP) increase and variation of flow structure that both suggest predisposed areas to atherogenesis and thrombus formation in different regions in ascending aorta, aortic arch and descending thoracic aorta.

**Electronic supplementary material:**

The online version of this article (10.1007/s10439-020-02448-6) contains supplementary material, which is available to authorized users.

## Introduction

Atrial fibrillation (AF) is the most common arrhythmia. It can exist in paroxysmal, persistent and long-standing persistent forms.^28^ AF normally occurs in adults, and the likelihood of occurrence roughly increases with increasing age.^57^ In the UK alone around 1,180,000 AF cases were recorded between 2015 and 2016. The statistical data for the same region for the period 2004 to 2016 shows that the incidence of AF tends to increase as the population becomes older.^8^ While AF has been considered as an independent risk factor, it occurs concomitantly with other diseases like hypertension and heart failure or can autonomously cause other types of cardiovascular diseases (CVDs) such as heart failure^6^ and stroke.^34^ Besides healthcare related issues, patients suffering from AF incur significant treatment costs,^45^ since the disease necessitates the long-term clinical treatment and follow-up.

Perhaps the most significant complication associated with AF is blood stasis inside the left atrium (LA) and formation of thrombus. Embolism of the thrombus can lead to distant organ ischaemia and infarction. In particular, cerebral embolism leads to a stroke. In a longitudinal study of participants from Framingham (known as the Framingham Heart Study),^62^ it was concluded that patients with AF are more vulnerable to ischaemic stroke and the condition worsens as the population become older. Additionally, a study by Camm *et al*.^10^ demonstrated that AF related strokes are severe. While stroke is considered one of the main consequences of AF, a recent study by Gómez-Outes *et al*.^19^ articulated that only a small proportion of deaths in AF population is because of ischaemic stroke; but the main reasons are heart failure, sudden death and myocardial infarction. Generally, discussion about AF effects is very challenging because it occurs in conjunction with other diseases; furthermore, the concomitant incidence of electrophysiological disorder, structural remodelling and flow changes during AF, make it a complicated disease.

One practical approach to explore in isolation the effect of various parameters and their impact on the disease is mathematical modelling of AF. Since AF stems from disorder in mechanical and electrical characteristics of the heart, mathematical modelling of electrophysiology and electromechanical behaviour of the heart during AF has been the focus of a significant body of research in recent years.^61^ However, to explore AF effects on haemodynamics of the cardiovascular system, lumped modelling and computational fluid dynamics (CFD) are two feasible techniques.

Using the lumped modelling approach for AF,^54^ two studies have been performed to explore AF effects on cerebrovascular circulation^55^ and its relevance to cognitive impairment.^4^ Similarly, further investigations on exercise tolerance during AF^5^ and the efficiency of the aortic and pulmonary valves^52^,^56^ have been accomplished. Recently, using a proposed multiscale approach,^21^ Scarsoglio *et al*.^53^ investigated AF effects on cardiovascular haemodynamics. Their findings clearly demonstrate that the arterial system cannot significantly damp AF effects; which thus remain as persistent perturbations with potential for adverse impact on the cardiovascular system.

Unlike 2D/3D CFD methods, lumped and one-dimensional approach cannot examine local variations of flow structure and associated haemodynamic metrics during AF. Therefore, employing 3D CFD approach, Choi *et al*.^12^ examined different aorta morphologies during AF-resulted strokes. The main outcome of their study emphasised that in cases with mild aortic arch (AoA) curvature, the possibility of stroke occurrence during AF increases up to three-fold comparing with the normal cardiac rhythm. One of the primary studies of intracardiac flow during AF was undertaken by Zhang *et al*.^66^ Using an idealised model of an LA they mainly examined the role of left atrial appendage (LAA) during AF. They demonstrated that during AF, the vortex structure changes and emptying of the LAA doesn’t take place appropriately, which can increase the possibility of thromboembolism. Koizumi *et al*.^35^ explored AF effects on LA haemodynamics using a patient-specific model. They evaluated two main biomarkers of AF, i.e. lack of atrial kick (AK) at late diastole and high frequency fibrillation (HFF). Their results suggested that both AF features influence blood flow and increase the possibility of blood stasis inside the LAA. In another effort by Otani *et al*.,^47^ effects of structural remodelling of LA due to AF on intra-atrial flow characteristics were examined. The study confirmed a mechanistic link between LA structural remodelling and thrombosis. Masci *et al*.^39^ improved the personalised CFD simulation of the intra-atrial flow during AF for risk stratification of stroke and therapy planning. Recently, Garcia-Isla *et al*.^18^ performed a sensitivity analysis on different configurations of LAA and pulmonary veins to quantify the risk of thrombus formation during AF.

In the context of AF, stroke is regularly postulated to be linked to intra-atrial clot, but it is less commonly considered that thrombus formation due to AF may also occur in the main aortic conduits. Given the literature about AF, its different aspects have been explored, both clinically and numerically, however, less attention has been paid to the downstream impact of AF on the circulatory system using 3D patient-specific geometries. In this study, four main consequences of AF—lack of AK, left atrial remodelling (LAR), left ventricular systolic dysfunction (LVSD) and HFF—are examined numerically to predict flow changes in the systemic circulation. To mimic four AF-associated defects, a lumped model for the left heart is employed, which produces the corresponding flow rate at the aortic root. Subsequently, the obtained flow rates are applied as the inflow to a patient-specific model obtained using 4D PC-MRI modality. Therefore, present study aims to investigate changes in haemodynamic metrics of aortic circulation, flow perfusion and genesis of vascular anomalies, specifically atherogenesis.

## Materials and Methods

### Magnetic Resonance Imaging Data Acquisition

In this study, aortic anatomical and 4D flow data were acquired for a 31-year-old healthy male volunteer using a 3T-Philips Achieva MRI scanner located at the NIHR Manchester Clinical Research Facility at Manchester Royal Infirmary, UK. The study was approved by the National Research Ethics Service (REC Ref 04/Q14002/11) and the subject gave informed consent. A high resolution T2-weighted structural scan was performed under breath-hold to extract morphological information; and a free-breathing, ECG gated, 4D phase contrast magnetic resonance imaging (PC-MRI) scan was used to extract 3D velocity data over 20 cardiac phases. Table 1 shows the parameters used for each scan. In Table 1, Scan 1 and Scan 2 refer to the flow and anatomy images, respectively.

**Table 1:** Scan parameters, MRI data.

Parameters	Scan 1 (PC-MRI—4D flow)	Scan 2 (T2W—anatomy)
Imaging matrix (pixel)	224 × 224 × 50	256 × 256 × 90
Plane resolution (mm^2^)	1.5625 × 1.5625	1.2466 × 1.2466
Slice thickness (mm)	2.5	0.9
No. of slices	50	100
Time increment (ms)	41.368	–
Number of phases in an *R*–*R* interval	20	1
Repetition time (ms)	3.2582	3.3178
Echo time (ms)	1.834	1.659
Flip angle (degree)	8	45
Velocity encoding (cm/s)	200	–

### Geometry

The anatomy was reconstructed from the data of a healthy volunteer. The geometry comprises ascending aorta (AA), AoA, descending aorta (DA), and the main branches including left coronary artery (LCA), right coronary artery (RCA), right subclavian artery (RSCA), right common carotid artery (RCCA), left common carotid artery (LCCA), and left subclavian artery (LSCA). To reconstruct the geometry, SimVascular image processing toolbox (Version 19.03.09),^37^ and CAD software, SolidWorks 2017 (SP 2.0) were used. More details about the geometry reconstruction have been provided in the Supplementary Materials.

### Governing Equations

In this study the blood flow was considered as an incompressible, homogenous, and Newtonian fluid. Findings show that for the large vessel, in healthy condition and in shear rates above 100 s^−1^ the blood behaves like a Newtonian fluid.^20^,^24^,^31^ Therefore, in this study the continuity equation along with Navier–Stokes equations were invoked, which are defined as follows:

1 $$\frac{\partial \rho }{\partial t} + \frac{\partial }{{\partial x_{i} }}\left( {\rho u_{i} } \right) = 0$$

2 $$\frac{{\partial \rho u_{j} }}{\partial t} + \frac{\partial }{{\partial x_{k} }}\left( {\rho u_{k} u_{j} } \right) = \frac{{\partial \sigma_{fij} }}{{\partial x_{i} }} + \rho f_{j}$$

In which $\rho$ is the density, equal to 1060 kg/m^3^ for the blood, *u*_*k*_ denotes fluid velocity components, *x*_*i*_ is the coordinate system, *f*_*j*_ denotes the body force per unit of volume, which is also equal to zero and $\sigma_{ij}$ is stress tensor that for the Newtonian fluid it can be defined as follows:

3 $$\sigma_{fij} = - \,p\delta_{ij} + \lambda \delta_{ij} \frac{{\partial u_{k} }}{{\partial x_{k} }} + \mu \left( {\frac{{\partial u_{i} }}{{\partial x_{j} }} + \frac{{\partial u_{j} }}{{\partial x_{i} }}} \right)$$

where *p* is the pressure, $\delta_{ij}$ is the Kronecker delta. Furthermore, $\mu$ is the first coefficient of viscosity (dynamic viscosity) and was taken 0.0035 Pa s for the blood, and $\lambda$ is the second coefficient of viscosity (volume viscosity) assumed to be zero for an incompressible flow.

Furthermore, to calculate the changes in blood perfusion throughout the aorta and its main branches during AF, the area average flow rate is integrated over a cardiac cycle through:

4 $$Q_{\text{total}} = \mathop \smallint \limits_{0}^{{t_{\text{cc}} }} \left( {{\iint }Q_{A} \left( t \right){\text{d}}A} \right){\text{d}}t$$

### Boundary Conditions (BCs)

**Figure 1:**
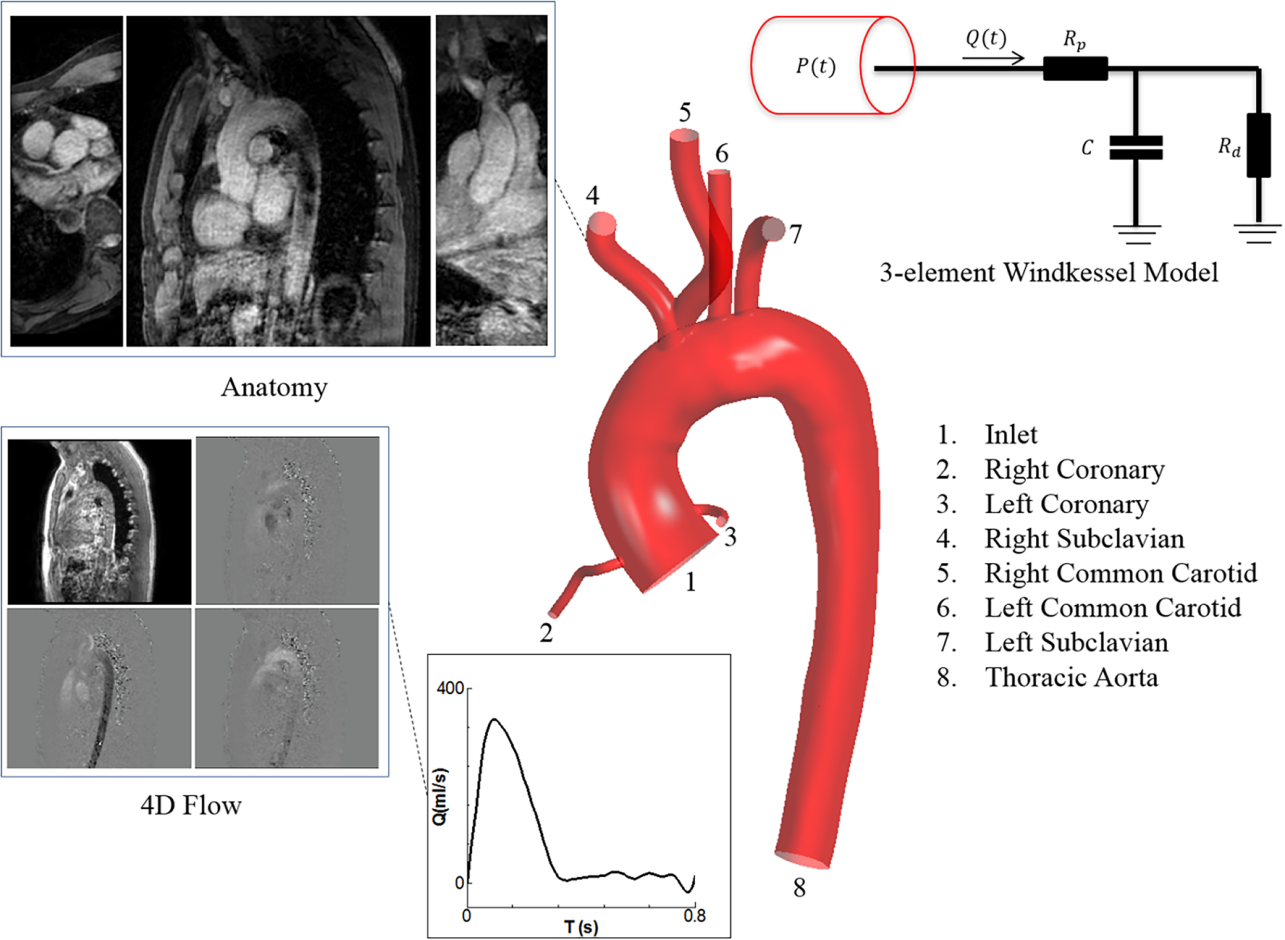
A schematic of model construction, from PC-MRI data acquisition (Anatomy and 4D flow data) to geometry reconstruction, and selected boundary conditions for the inlet and outlets.

The model consists of one inlet and seven outlets as displayed in Fig. 1. For the inlet a subject-specific velocity waveform extracted from the PC-MRI data was prescribed as a plug flow. In this study the aorta was assumed to be rigid, which is a reasonable compromise of accuracy, data availability and computational cost.^9^ Furthermore, at the wall vicinity no-slip condition was applied. For all the outlets, three-element Windkessel (RCR) model was used, which has been demonstrated an ample 0D–3D coupling.^33^,^40^,^48^ Additionally, this model can compensate the absence of the wall elasticity.^50^ The RCR model and its corresponding parameters are defined in the Supplementary Materials.

### Compact Lumped Model for the Arterial Circulation

For the parametric study of AF effects, the left heart function was mimicked using the model proposed by Simaan *et al*.,^58^ which considers the LA, mitral valve (MV), left ventricle (LV) and aortic valve (AV) that were coupled with the aorta and systemic circulation. To study different phases inside the LA, i.e. reservoir, conduit and booster pump, the LA compliance has been modified as a time-variant parameter. The circuit can produce corresponding inlet waveform for the left ventricular outflow tract (LVOT) as the left heart parameters change, so the resultant waveform can be applied as the inlet BC at aortic root to investigate flow distribution/perfusion during AF. More details are provided in the Supplementary Materials.

### Four AF Characteristics

Since AF directly impacts the mechanical and functional characteristics of LA and LV, it therefore affects the flow at LVOT. In the following, each AF defect and the corresponding parameter used to mimic specific abnormality is introduced as follows:

1. **Lack of AK** the AK usually occurs at late diastole to eject remaining blood into the LV. As the atrium loses its active contraction, the flow toward the LV reduces.^2^ To mimic this abnormality, it can be reflected through the LA elastance by assuming that it remains constant during a cardiac cycle.^54^ The comparison was made for six different LA constant elastances (ELAC) in different orders of magnitude with the values of 0.002, 0.02, 0.2, 2, 20 and 200 mmHg/mL, which correspond to ELAC1 to ELAC6, respectively.
2. **LAR** can result from chronic AF,^46^ owing to the genesis of fibrosis substrate and larger LA size.^36^ In this study it is postulated that LAR is associated to the alteration of the LA compliance. As compliance is inversely proportional to elastance, six different LA elastances (ELA) were used. Numeric values for (ELAmin1,ELAmax1) to (ELAmin6,ELAmax6) are (0.002,0.003), (0.02,0.03), (0.2,0.3), (2,3), (20,30) and (200,300) mmHg/mL, respectively. Noting that the baseline values for the normal LA elastance was taken (0.2, 0.3) mmHg/mL, which is in a same order adopted by Scarsoglio *et al*. 54.
3. **LVSD** is another side effect of AF which appears as instantaneous or permanent change in LV function.^11^ To simulate this condition, it was assumed that the LV elastance (ELV) changes, and therefore variations are compared for five different maximum elastances (ELV_max_) to mimic its systolic dysfunction. The chosen values for ELV1_max_ to ELV5_max_ are 0.3, 0.5, 1, 1.5 and 2 mmHg/mL, respectively, while ELV_min_ for all the cases was kept constant and it is equal to 0.05 mmHg/mL.^54^,^59^ The normal value in this case is (ELV_min_ = 0.05,ELV_max_ = 2)mmHg/mL.
4. **HFF** heartbeats in a patient with AF normally ranges between 100 and 175 bpm. To investigate this feature of AF, three different cases, i.e. 75 (normal case), 100 and 150 bpm were chosen,^13^ while it was assumed that the diastolic volume remains constant at different frequencies.

The baseline values of the left heart model are presented in the Supplementary Materials, and they are indicated as normal (*N*) in the figures. In this study the pattern of flow waveform at different cycles was assumed to remain unchanged, and the irregularities were ignored. Indeed, given regression analyses, which is based on preceding (RR_p_) and pre-preceding (RR_pp_) interval of waveforms during AF, it has been confirmed that for *RR*_p_/*RR*_pp_ = 1, the cardiac parameters reflect average values during AF.^59^,^60^

### Numerical Method

The continuity and Navier–Stokes equations were discretised numerically using ANSYS-CFX 19.0, which uses finite volume method. The advection terms were discretised using high-resolution method—this scheme uses either 1st order or 2nd order accuracy in space depending on flow field condition to impose the boundedness condition. Moreover, a 2nd order backward Euler scheme was invoked to discretise the time derivative. The convergence criteria for the simulation are based on root mean square (RMS) of residuals of mass and momentum equations and were set to 10^−6^.

To implement Windkessel model for all the outlets, the differential equations were discretised implicitly using 1st order backward Euler scheme. Furthermore, for the inlet, a Fourier series with eight harmonics was fitted to the data obtained from 4D PC-MRI, using the least square method. Finally, the set of first order ordinary differential equations (ODEs) obtained from the left heart lumped model were solved using a fourth-order Runge–Kutta method.

To obtain a converged solution which is independent of grid size, four different grid sizes were examined. The computational domain consists of tetrahedral elements, which are accompanied with five prism layers for a proper treatment of near wall region. Mesh sensitivity analyses showed that the computational domain with 6.6 million elements is fine enough to capture all the flow features precisely. Furthermore, for a stable solution, timestep size of 0.1 ms was chosen and the simulation was performed for four cardiac cycles to get fully converged temporal solution. More details are provided in the Supplementary Materials.

## Results

### Validation

**Figure 2:**
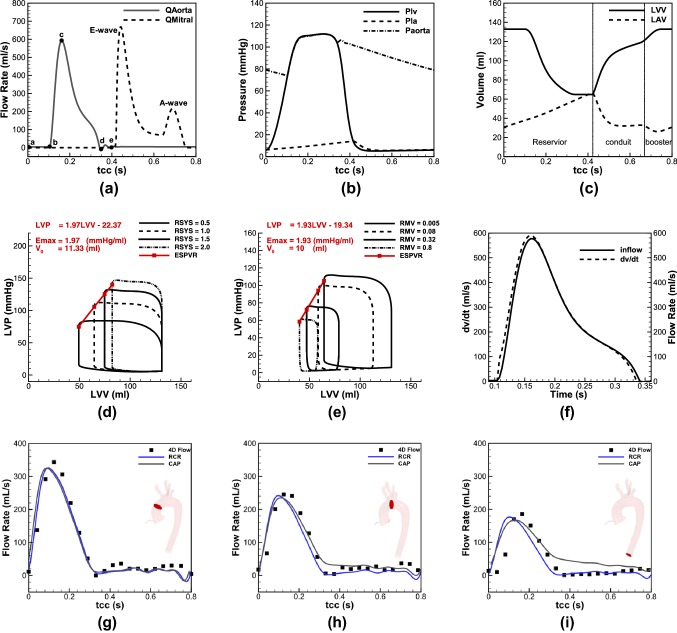
Validation of the left heart lumped model; (**a**) flow rate at the LVOT, (**b**) pressure waveform of the LV, LA and aorta, (**c**) volume changes of the LA and LV, (**d**) ESPVR for different afterload conditions in a constant end diastolic volume, (**e**) ESPVR for different preload conditions, (**f**) volume changes of the LV, and aortic flow rate; validation of CFD against *in-vivo* (4D PC-MRI data) (**g**) ascending aorta, (**h**) aortic arch (between brachiocephalic artery and LCCA), (**i**) DTAO.

In this section two sets of validation have been presented. To show the robustness of the left heart model, Fig. 2 represents the waveforms of the flow rate, pressure, volume changes and LV function using the parameters employed in study by Simaan *et al*.,^58^ which was validated against the clinical data (Figs. 2a–2e). Figure 2a displays flow rates across the AV and MV and shows the key moments in a cardiac cycle. Figure 2b displays pressure waveforms of aorta, LV and LA. For a normal heartbeat of 75 bpm the model can successfully predict systolic and diastolic pressures around 113 and 75 mmHg, respectively, and CO of 5.12 L/min, which are in physiological ranges reported for healthy individuals.^23^ Furthermore, LA pressure is successfully predicted to vary between 10 and 20 mmHg, which has a good agreement with *in-vivo* measurements.^44^ In Fig. 2c the LV volume changes between 60 and 140 mL, while the LA volume alters between 20 and 60 mL, which are well located within the range measured amongst adults.^22^,^25^ Figures 2d and 2e plot left ventricle pressure (LVP) vs. left ventricle volume (LVV) for afterload and preload, respectively; a linear relation for the end systolic pressure volume relation (ESPVR) is recovered as noted in previous studies.^58^ Finally, Figure 2f shows the coincidence of the volume changes of LV and aortic flow when the AV is open, which is in agreement with the clinical measurements.^25^

To evaluate the accuracy of the CFD model, the resulting flow rates from the numerical modelling are compared against the data obtained from 4D PC-MRI. To extract *in-vivo* data from different cross sections, the flow data—known as phase and magnitude images—were imported to an open source code, *Segment*^26^ and the flow rate at each particular plane was extracted individually and collected to obtain the total value. The comparison was made for three sections in AA, AoA (between brachiocephalic trunk and LCCA) and descending thoracic aorta outlet (DTAO), as shown in Figs. 2g–2i. The MRI data were taken from twenty phases in a cardiac cycle, while the numerical data was obtained for two different assumptions for the outlet BCs to test the effects of flow downstream; therefore, constant average pressure (CAP), which neglects the peripheral arteries resistance and RCR model that includes this effect. Furthermore, in Table 2 the mean flow rate of different cases is compared. Results showed that the RCR model can successfully predict flow waveforms and their mean values (comparing with the *in-vivo* data), while the CAP predicts flow rate less accurate, particularly at distal region with respect to the aortic root.

**Table 2:** Average flow rate across three defined sections.

	*Q*_avg_ (mL/s)		
Case	AA	AoA	DA
4D PC-MRI (*in-vivo*)	90.10	70.92	49.58
CAP (CFD)	85.54	75.13	66.70
RCR (CFD)	83.65	61.30	44.76

### Cardiac Metrics

In this section the main cardiac metrics including pressure, flow rate, cardiac output (CO), stroke volume (SV) and ejection fraction (EF) are examined during four AF defects.

**Figure 3:**
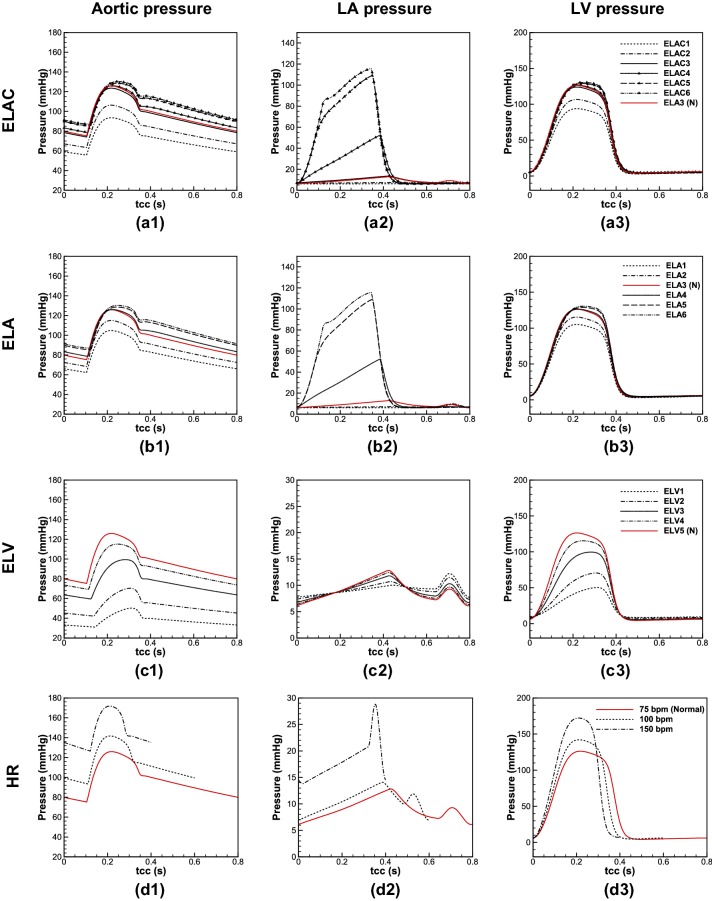
Pressure waveforms at: (**a.1**) aorta, (**a.2**) LA, and (**a.3**) LV, in Lack of AK; (**b.1**) aorta, (**b.2**) LA, and (**b.3**) LV in LAR; (**c.1**) aorta, (**c.2**) LA, and (**c.3**) LV in LVSD; (**d.1**) aorta, (**d.2**) LA, and (**d.3**) LV in HFF (all the pressure waveforms are shown for a cardiac cycle).

In Figs. 3(a.1)–3(a.3) and 3(b.1)–3(b.3), aortic, LA and LV pressures are depicted, respectively, for different ELAC and ELA. Comparing these models, meaningful differences can be seen in aortic and intracardiac pressures (LA and LV pressures) for the values below and in normal range, however, as the elastance increases above the normal value (ELA3), the difference becomes negligible. Figures 3(c.1)–3(c.3) show pressure changes due to the LVSD. As displayed, significant changes occur for aortic and intracardiac pressures. Moreover, for the lower values of ELV, the peak pressure for the aorta and LV takes place later. Finally in Figs. 3(d.1)–3(d.3) the pressures are shown in different HR. Results show that increase in number of beats during AF increases the intracardiac and aortic pressures significantly, and it changes slightly the waveform patterns.

**Figure 4:**
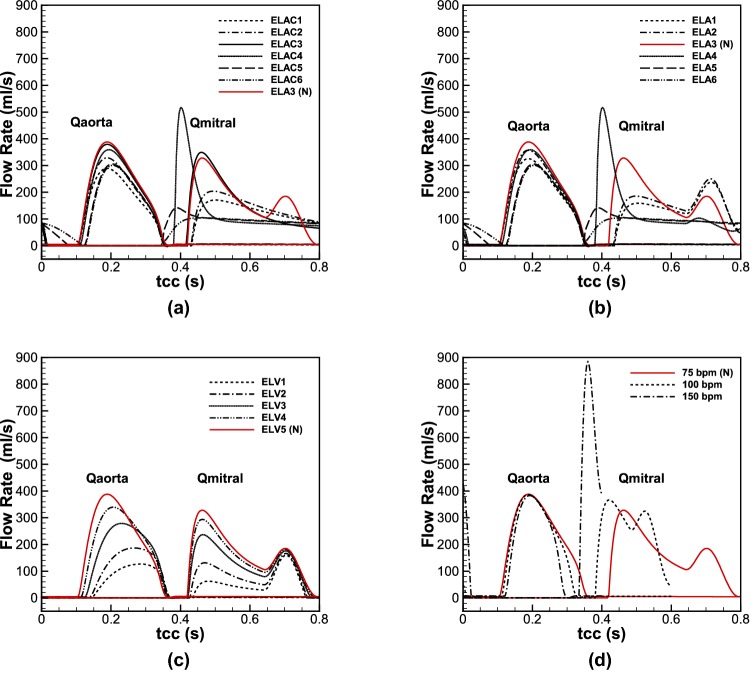
Flow rates at the AV and MV; (**a**) lack of AK, (**b**) LAR, (**c**) LVSD, and (**d**) HFF.

Figure 4 shows the flow passes across the AV and MV during different AF-related defects. In Figs. 4(a) and 4(b) the aortic and mitral flows are compared for different elastances in absence and presence of AK, respectively. Like the pressure waveforms, the flow rate difference for the cases with and without AK is observable for the lower values of elastance (ELA1–ELA3/ELAC1–ELAC3), however, for the larger values, the difference becomes negligible.

Figure 4c displays different flow waveforms across the MV and AV due to the changes in LV elastance. The results demonstrate that LVSD is accompanied with flow reduction from the LA to the LV during the passive contraction, which consequently reduces flow at LVOT. Furthermore, as the ELV decreases, the peak of aortic flow waveform takes place later, which is in accordance of the pressure waveform described earlier. In Fig. 4d the flow rates are shown for different Heart Rates (HRs). By increasing the HR, the blood flow from the LA to the LV reduces, which decreases aortic flow as well. Furthermore, a significant raise occurs for the peak flow of MV at 150 bpm.

**Figure 5:**
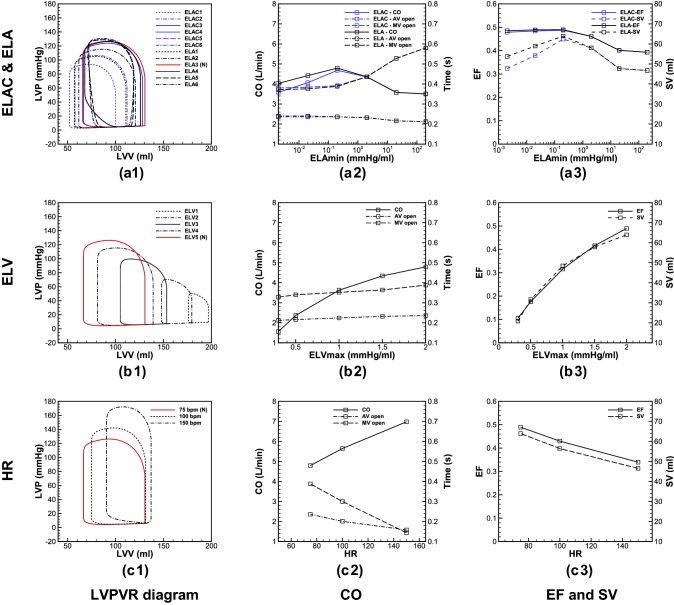
LVPVR loop during (**a.1**) lack of AK and LAR, (**b.1**) LVSD, and (**c.1**) HFF; CO and duration in which AV and MV remain open during (**a.2**) lack of AK and LAR, (**b.2**) LVSD, and (**c.2**) HFF; changes of EF and SV per beat during (**a.3**) lack of AK and LAR, (**b.3**) LVSD, and (**c.3**) HFF.

To explore the heart function, Fig. 5 displays LV pressure–volume relation (LVPVR), CO, SV and EF. On LVPVR diagrams, three main characteristics of LV functionality can be detected, which are preload, afterload and LV contractility.^17^ Figure 5a.1 compares LVPVR diagram for different LA elastances with and without AK. Results show that for the LA elastance below ELA3, lack of AK causes a decrease in preload and afterload, which lead to SV and CO reduction as shown in Figs. 5a.2 and 5a.3. For the larger elastances (ELA4–ELA6) the AK effect gradually disappears, and no differences can be observed in cardiac metrics between ELA and ELAC. Figures 5b.1–5b.3 illustrate changes in cardiac metrics during LVSD. The outcomes show that reduction in LV systolic function is accompanied with increase in preload and afterload, while the LV contractility diminishes drastically, and causes SV, EF and CO decrease. Figures 5c.1–5c.3 display cardiac metrics in various HRs. Considering LVPVR diagram, it reveals that as the HR increases, preload changes slightly, however, afterload undergoes more significant changes, while the contractile of the LV does not affect visibly. As a result, CO increases, however, reducing in time through which the valves remain open, SV and EF decrease significantly.

### AF Effects on Flow Distribution Throughout the Aortic Circulation

In order to systematically assess AF related changes in a qualitative manner, standard haemodynamic metrics are invoked. Time-averaged wall shear stress (TAWSS), oscillatory shear index (OSI) and TAWSS gradient (TAWSSG) are employed to consider the mean behaviour of WSS, occurrence of reversed flow and local variation of WSS, respectively.^30^,^51^ Furthermore, the ratio of OSI to TAWSS, known as endothelial cell activation potential (ECAP),^15^ which shows thrombogenic-prone regions through the arterial system. Previous studies have shown that for the values of TAWSS less than 0.36 Pa, monocytes are prone to adhere to endothelial cells which could lead thrombogenesis.^16^,^63^ Moreover, high OSI values indicate disturbed flow region at the vascular wall, where the WSS vector drastically change its direction over the cardiac cycle. Therefore, ECAP value around 1.4 is considered as the threshold value for thrombogenesis. Moreover, to improve visualisation of the unsteady flow features, iso-surfaces of the *Q*-criterion are used.^27^

As discussed in the previous section, lack of AK, LAR, LVSD and HFF all alter flow passes across AV. Therefore, changes in inflow boundary condition during AF would influence aortic haemodynamics. To this aim ECAP and TAWSSG in a cardiac cycle, and vortex strength and velocity contours at systolic peak are examined. To compare haemodynamic variations of the defects with each other, two cases are presented for each anomaly, which are compared against the baseline values. ELA1 and ELA6 for the LAR, ELV1 and ELV3 for the LVSD, and 100 and 150 bpm for the case of HFF.

**Figure 6:**
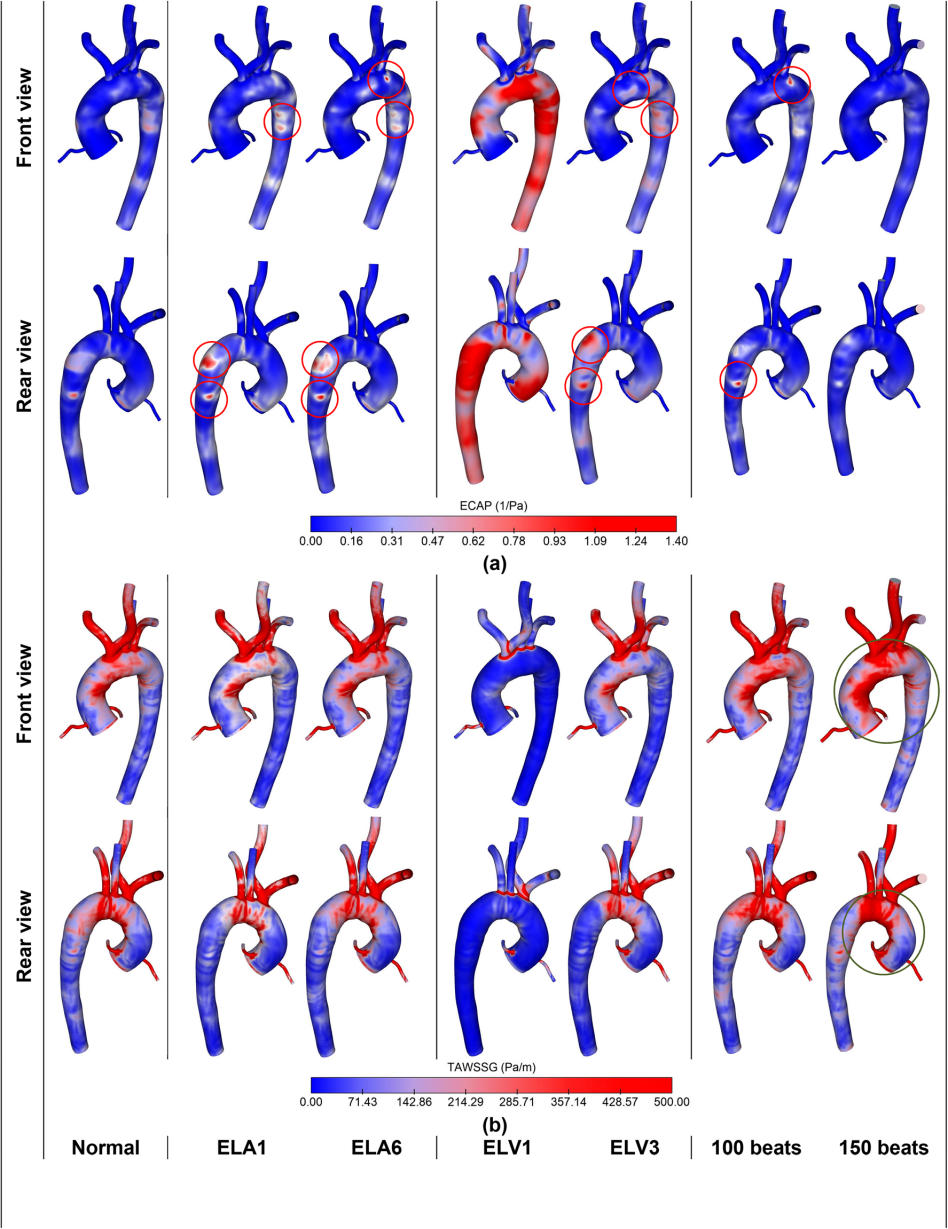
Comparison of haemodynamic metrics in aortic circulation in normal and AF-associated defects: (**a**) ECAP = OSI/TAWSS, and (**b**) TAWSSG (for each AF-related defect two cases are shown).

Results are displayed in Fig. 6 for three different LA compliances. From ELA1 to ELA3, the flow rate at LVOT increases, which lowers ECAP at the AA, AoA, DA. For the ELA values higher than ELA3, LA loses its active contraction, and so the flow across the LVOT diminishes, which is accompanied by higher ECAP.

Patterns of TAWSSG within the considered range do not change significantly, however, the variations become more visible for the larger vessels including aortic artery, LCCA, RCCA, LSCA and RSCA, while for the LCA and RCA this variation is less significant. Furthermore, given the diameter of the artery, and across the bends and curvatures, TAWSSG increases.

In LVSD, decrease in LV elastance reduces the flow output, which significantly influences ECAP and TAWSSG. For ELV of 0.3 mmHg/mL (ELV1), ECAP crosses the threshold value of 1.4 mmHg/mL, while for ELV3 it marginally exceeds the limit. Therefore, the susceptible regions in ELV1 and ELV3 are the aortic root, AoA and DA. Moreover, for ELV1 the ECAP grows considerably at supra-aortic branches.

TAWSSG also undergoes significant changes. Indeed, for low ELV, TAWSSG decreases since less blood flows to the aortic circulation; while it increases as the ELV increases. The results demonstrate that for low values of ELV, TAWSSG decreases along with decrease in TAWSS (contours of TAWSS and OSI are presented in the Supplementary Materials).

Finally, the fourth column in Fig. 6 demonstrates ECAP and TAWSSG during high HR. The results show that in the high HR, ECAP decreases, while TAWSSG increases, conversely. This increase is more pronounced at the AA and AoA.

**Figure 7:**
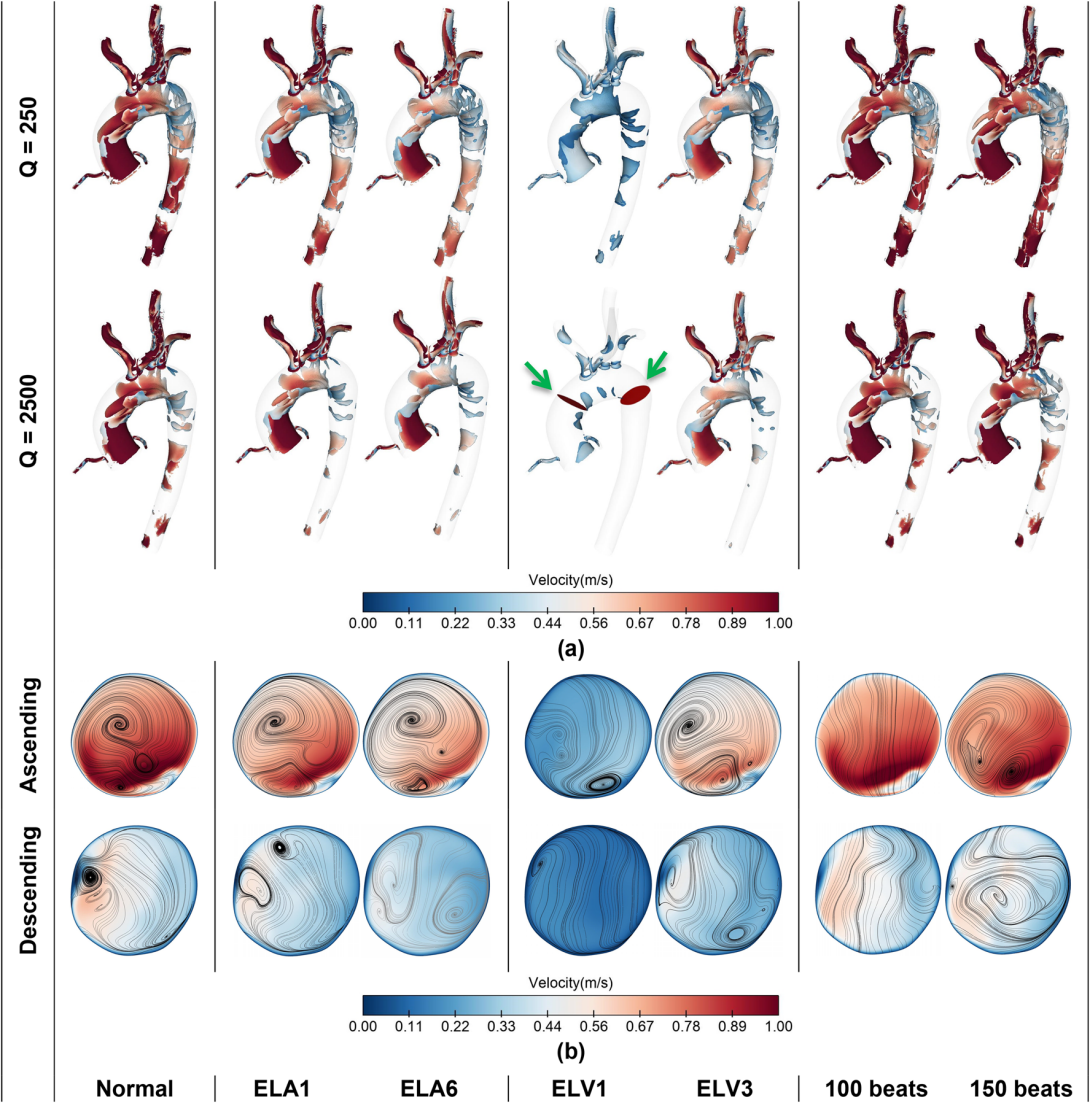
(**a**) vortex intensity for two iso-surface values using Q-criterion; (**b**) velocity contours and in-plane streamlines at two planes across the AA and DA (shown by green arrows); (the results are compared between normal aortic flow and AF-related defects).

Figure 7 shows the flow structure in terms of velocity, streamline and vortex strength. To visualise vortex strength, Q-criterion is used, and the results have been depicted for two iso-surface values of 250 and 2500. Additionally, velocity contours at two different cross sections, in the AA and DA are displayed, which also contain planar streamlines. All the results in Fig. 7 are illustrated at systolic peak.

The results are shown for the LAR, LVSD and HFF and compared with the baseline values. Figure 7a shows that LAR does not affect vortex strength significantly, while it could change the velocity magnitude and vortex arrangement, specifically at the DA as shown in Fig. 7b.

In contrast with LAR, LVSD can strongly affect aortic flow distribution. During severe systolic dysfunction (ELV1), the LV produces very poor inflow waveform, which diminishes vortex strength and creates poor vortex core regions at the AA and nearly uniform flow at the DA. By increasing ELV, for ELV3 the vortex strength increases, and the flow develops two vortices at the AA with lower intensity, while the flow is partially disturbed at the DA.

Furthermore, the results for higher HR, i.e. 100 and 150 bpm show that they do not change the vortex strength meaningfully, however, for the highest HR—150 bpm in this study—the flow tends to form stronger vortices. Worth mentioning that for the mid-value of HR, the flow at systolic peak does not form vortex at the AA and DA.

### Blood Perfusion

**Figure 8:**
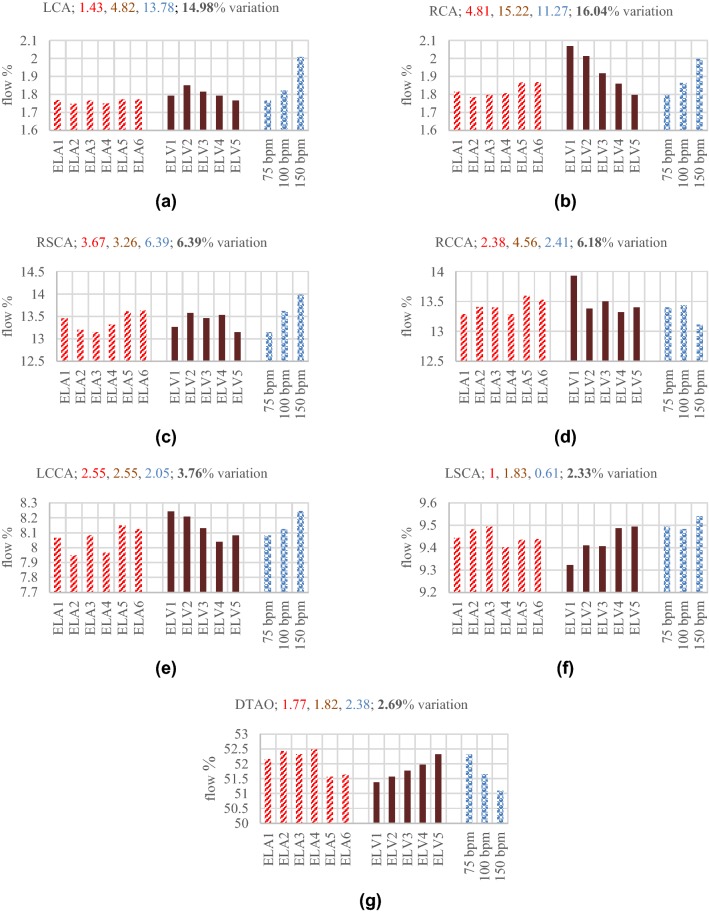
Flow percentage goes through each branch during various AF anomalies. For each case the flow percentage is evaluated based on mean flow rate at the inlet. Each number at top of the chart denotes the flow variation among different cases of particular AF defect; first number (red) for LAR, second number (crimson) for LVSD, third number (blue) for HFF, and finally fourth number (black) shows the total flow changes among different AF anomalies. (**a**) LCA, (**b**) RCA, (**c**) RSCA, (**d**) RCCA, (**e**) LCCA, (**f**) LSCA, and (**g**) DTAO.

In this section changes in blood perfusion throughout the aorta and its main branches during AF are investigated. Figure 8 displays blood perfusion in different branches of the considered aorta. The figure shows percentage flow through each outlet, and for each outlet, the perfusion variations are displayed for LAR, LVSD and HFF during AF.

The results demonstrate that among six sets of the LA elastance, ELA3 produces the largest flow rate, while deviation from ELA3, which is considered as the normal elastance, leads to lower flow rates at LVOT. Figure 8 shows the flow percentage through each branch for the various ELAs. The results illustrate that as the flow rate decreases; the general trend for percentage flow through the coronary arteries is incremental, while it is decremental for the DTAO. Furthermore, given the location of supra-aortic arteries the flow percentage changes in different LA elastances. For the LCCA and RCCA the patterns are similar to the coronary arteries, however, for the RSCA and LSCA, no regular pattern can be observed.

During LVSD as clarified in Fig. 8, the aortic flow decreases as the ELV becomes smaller (LV contraction capability reduces). The results show that as the ELVmax increases and becomes closer to the normal LV elastance, the percentage flow through the coronary arteries decreases, while the flow at the DTAO increases. Moreover, there are significant flow variations in the RCA (15.22%) and LCA (4.82%) in different ELVmax. Flow variations in the supra-aortic arteries does not follow a regular pattern, however, in the LCCA and RCCA show a descending trend as flow increases at the LVOT, while in the LSCA reveals an ascending trend.

In case of HFF, by increasing the HR, the flow percentage through the coroanary arteries increases (13.78% for the LCA and 11.27% for the RCA), while it decreases for the DTAO. Furthemore, the LCCA and RSCA reveal an increasing trend, and in general, flow in the RCCA tends to decrease, while for the LSCA, it increases. In summary, during AF since the flow at LVOT reduces, the overall perfusion decreases, while the flow percentage alters comparing with the normal condition.

## Discussion

In this study four common AF attributes (lack of AK, LAR, LVSD and HFF) on aortic flow distribution are examined. The pressure at the aortic root, LV and LA, the flow rate across the AV and MV were considered. Furthermore, other metrics including LVPVR, SV, EF, CO, ECAP, TAWSSG, vortex intensity and structure were examined.

Using different sets of elastance for the LA, AK and LAR effects were studied. The results showed that as the compliance of the LA decreases, the intra-cardiac and aortic pressure increases. This increase is much more severe for the LA, which imposes massive stress on it. Furthermore, in lower compliance—in reality it happens because of fibrogenesis—the atrium loses its active contraction attributes, which is in accordance with clinical reports.^1^ Consequently, the preload decreases for any compliance deviation from the normal value; however, the afterload becomes less than the normal value for the more compliant atrium, while it exceeds the normal value as the atrium becomes stiffer. Therefore, the SV and CO decreases, while EF does not change significantly. However, for the less compliant LA once it loses the AK feature, EF declines meaningfully.

The overall changes in LA compliance results a flow reduction across the MV and AV. Furthermore, considering blood perfusion, flow percentage through the RCA and LCA increases, while it decreases at the DTAO. Noteworthy to mention that the slopes of increase and decrease are marginally higher for the less compliant atrium. Therefore, lowering in flow passage across the LVOT leads to ECAP increase in some spots of the AoA and DA, which suggests thrombogenesis hazard. Moreover, changes in the LA elastance—as a result of either lack of AK or LAR—slightly decreases velocity magnitude and alters vortex structure because of flow reduction.

LVSD is another common defect occurs during AF. During severe dysfunction, the aorta, LV and LA experience significant pressure drop. However, once the AV closes and MV opens, the LA and LV pressures rise slightly for the lower LV elastance (more severe dysfunction) that imply higher stresses on the LA and LV. Therefore, by decreasing in LV functionality, both preload and afterload increase, while the contractility decreases significantly that all result a drastic reduction of CO, SV and EF. Moreover, current findings suggest that during LVSD, EF is correlated negatively to the ratio of LV end systolic pressure to SV (ESP/SV), while it is correlated positively to the ratio of LV end systolic pressure to LV end systolic volume (ESP/ESV). Similar conclusions were taken through a beat-by-beat analysis on seven AF patients by Munitinga *et al*.^43^ (the comparisons are presented in the Supplementary Materials).

Weak LV performance lowers blood flow across the MV and AV. Noteworthy to mention that the main flow reduction across the MV occurs during the passive LA contraction, while it slightly reduces during its active contraction. Furthermore, considering the blood perfusion, it explains that decrease in LV systolic function alters flow percentage through proximal and distal arteries in a way that it rises at the RCA and LCA, while it reduces at the DTAO.

Considering haemodynamic metrics in severe LVSD, it reveals observable changes in vortex structure and patterns that are reflected as a drastic decrease in TAWSS, and emergence of reversed flow (OSI increase) at some regions; therefore, the concomitant effect is that the ECAP crosses the threshold limit of 1.4. Therefore, for ELV1 the possibility of thrombogenesis increases at the AA, AoA and DA. However, as the LV recovers its normal function, ECAP decreases, so it reduces the thrombogenesis hazard.

One of the frequent AF features is HFF in which the HR undergoes up to three-fold increase. Additionally, HFF is accompanied with irregular HR at different beats,^14^ which was neglected in this study. Current findings showed that the aortic and intra-cardia pressures increase significantly. The obtained average LA pressure is around 20 mmHg, which has been observed among patients with persistent AF.^64^ Since the end diastolic volume is assumed to remain constant, therefore, the preload and the LV contractile are unchanged, while the afterload increases for the higher HR. Also, since HR irregularity is ignored—the condition occurs during atrial flutter—CO increases, while the SV and EF reduce significantly as concluded by Anselmino *et al*.^3^

Therefore, by increasing HR, despite negligible changes at aortic peak flow, however, the blood finds a little time to flow through the AV and decreases the circulatory perfusion. Like the other AF-related defects, during abnormal HR, the flow percentage through the RCA and LCA increases, while it decreases through the DTAO. Considering flow structure inside the aortic conduit, at HR = 100 bpm, vortices do not form at the AA and DA during the systolic peak, whereas for HR = 150 bpm, the vortices at the AA and DA are retrieved with higher intensities. In overall, decrease in ECAP during HFF, it decreases thrombus formation due to fatty substance adhesion; in contrast the critical increase in TAWSSG enhances the possibility of luminal lesions and damage on endothelia cells, which increases thrombogenesis risk.

In Table 3, the key findings of this work have been presented qualitatively. The results suggested that abnormal aortic flow significantly alters cardiac metrics and haemodynamic parameters. Consequently, the overall impact is an increase in the possibility of plaque formation, specifically at the AoA and DA. This possibility was emphasised by Blackshear *et al.*^7^ at DA. Accordingly, arterial stenosis and subsequent rupture, increase stroke threat as a consequence of embolus movement toward the carotid arteries. To establish a mechanistic link between the AF-related defects and their associations to the stroke, more investigations on different aortic morphologies are recommended.

**Table 3:**
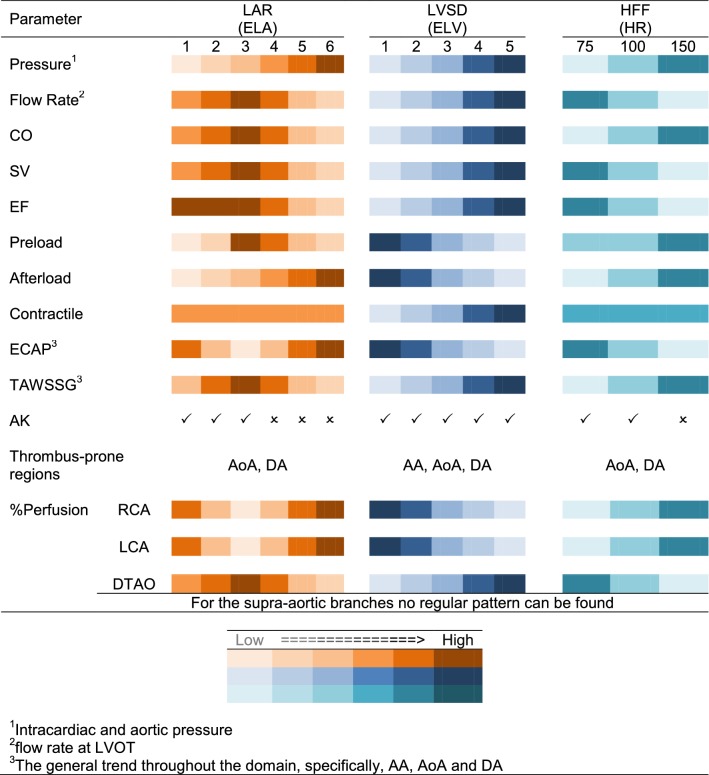
The trend of variation of each cardiac/haemodynamic metric in various AF defects (a qualitative summary of the key findings).

## Limitations

The present study has some limitations should be improved in future studies to address AF anomalies more precisely.

- The current work targeted a parametric study of an isolated AF-associated defects on aortic flow circulation. However, more precise outcome would be obtained if AF patient-specific inflow data was available, i.e. both image-based flow rate and velocity profile. In fact, it has been shown that the image-based subject-specific velocity profile could change haemodynamic metrics, particularly amongst patients and at AA and AoA.^42^,^49^,^65^
- In this study, the RCR Windkessel model was used for all the flow outlets, however, more accurate results will be obtained using the lumped model for the coronary arteries.^32^,^38^
- The flow is assumed to be laminar, however, it has shown that it tends to become turbulent, particularly at AA and AoA.^41^
- Another factor might hinder the accuracy of the result is the rigid wall assumption. The wall compliance can be included either as an interaction of the blood and vessel or by obtaining the history of deformation. The former requires subject-specific constitutive data, while the latter requires different morphological states in a cycle. These two models have been employed in a number of studies, however, they have some drawbacks (like lack of suitable clinical data and computational burden) that should be amended.^9^,^29^,^50^

## Electronic supplementary material

Below is the link to the electronic supplementary material.

Supplementary material 1 (DOCX 1764 kb)

## Abbreviations

AA: Ascending aorta
AF: Atrial fibrillation
AK: Atrial kick
AoA: Aortic arch
AV: Aortic valve
BC: Boundary condition
CAP: Constant average pressure
CFD: Computational fluid dynamics
CO: Cardiac output
CVD: Cardiovascular disease
DA: Descending aorta
DTAO: Descending thoracic aorta outlet
ECAP: Endothelial cell activation potential
EF: Ejection fraction
ESPVR: End systolic pressure volume relation
HFF: High frequency fibrillation
HR: Heart rate
LA: Left atrium
LAA: Left atrial appendage
LAR: Left atrial remodelling
LCA: Left coronary artery
LCCA: Left common carotid artery
LSCA: Left subclavian artery
LV: Left ventricle
LVOT: Left ventricular outflow tract
LVP: Left ventricular pressure
LVPVR: LV pressure–volume relation
LVSD: Left ventricular systolic dysfunction
LVV: Left ventricular volume
MV: Mitral valve
OSI: Oscillatory shear index
ODE: Ordinary differential equation
PC-MRI: Phase contrast magnetic resonance imaging
RCA: Right coronary artery
RCCA: Right common carotid artery
RMS: Root mean square
RSCA: Right subclavian artery
SV: Stroke volume
TAWSS: Time-averaged wall shear stress
TAWSSG: TAWSS gradient
