# Supplementary material for: Numerical Study of Atrial Fibrillation Effects on Flow Distribution in Aortic Circulation
Source: Ann Biomed Eng. 2020 Jan 14;48(4):1291–308. doi: 10.1007/s10439-020-02448-6 (PMC7089914; doi:10.1007/s10439-020-02448-6)
Supplement: Supplementary file 1 — Supplementary material 1 (DOCX 1764 kb) [file 10439_2020_2448_MOESM1_ESM.docx]

**Supplementary Materials**

# Grid Network

To create an accurate computational domain, four grid networks with different densities were created. The whole domain was meshed using tetrahedral cells with five prism layers at the wall boundary. Figure 1 shows the mesh.

| 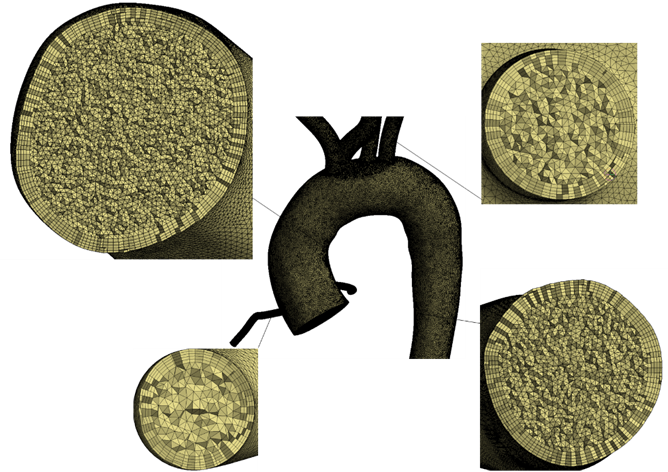 |
| --- |
| Figure 1. Grid network with 6.6 million cells |

Furthermore, mesh sensitivity analysis was performed and finally a domain with the total number of around 6.6 million cells returned mesh independent results. In Figure 2 velocity and total pressure at the aorta’s centreline are compared for different sets of mesh densities. Similar comparisons were made at different branches (not shown here).

|  |  |
| --- | --- |
| (a) | (b) |
| Figure 2. Different mesh densities at the aortic centreline, (a) velocity, (b) total pressure. | |

# Constant Average Pressure

Constant Average Pressure (CAP) is calculated for all the outlets using the mean value of systolic and diastolic pressures for the healthy volunteer. The systolic and diastolic pressures were 120 mmHg and 80 mmHg, respectively. Using these two values the CAP for all the outlets was calculated as follows:

| $CAP=\frac{P_{systole}+2P_{diastole}}{3}$ | (1) |
| --- | --- |

# Geometry Reconstruction

The raw MRI images were exported as classical DICOM format. Thereafter the DICOM images were imported in SimVascular (Version 19.03.09) for the initial processing including seed emplacement, creation of path-line and segmentation. Subsequently the path-lines and segmented cross sections were imported as a set of points in SolidWorks 2017 (SP 2.0) for lofting and reconstruction of the final geometry. Figure 3 displays the pipeline employed for the geometry reconstruction.

| 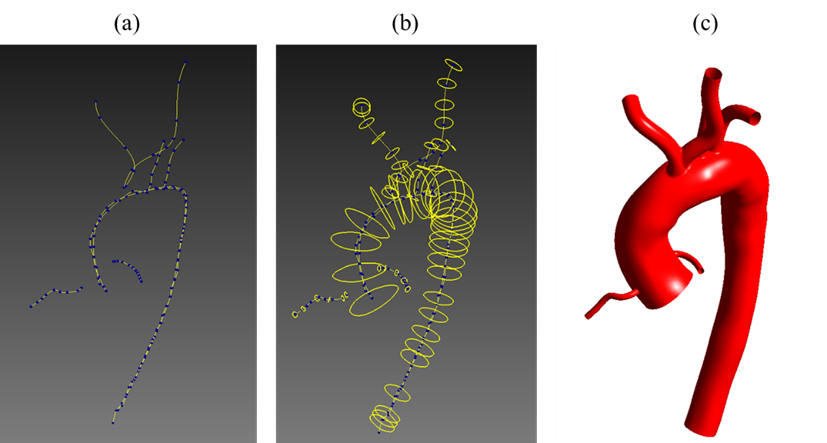 |
| --- |
| Figure 3. (a) seed points and path-lines, (b) segmentations, (c) lofted segments |

# Curve-Fitting Method

In order to apply a pulsatile flow as the inlet boundary condition in ANSYS-CFX, the obtained 4D flow data needs to be estimated as a mathematical equation to be usable for the numerical simulation. Given the shape of the cardiac pulses, Fourier series is a suitable function for performing curve-fitting through 4D flow data. Least Square Method (LSM) was used for doing a proper fitting using the Fourier series as the base function.

Fourier series can be defined as follows:

| $F\left( x \right)= a_{0}+\sum_{n=1}^{N} (a_{n}cos\omega_{n}x+b_{n}sin\omega_{n}x), \omega_{n}=\frac{2\pi n}{T}$ | (2) |
| --- | --- |

To fit the considered function through the set of data as ($x_{i},y_{i};i=1,m$), using the LSM, it can be expressed as:

| $E= \sum_{i=1}^{m} \left( y_{i}-F\left( x_{i} \right) \right)^{2}=\sum_{i=1}^{m} \left( y_{i}-(a_{0}+\sum_{n=1}^{N} (a_{n}cos\omega_{n}x_{i}+b_{n}sin\omega_{n}x_{i})) \right)^{2}$ | (3) |
| --- | --- |

To obtain a perfect fitting, coefficient *a_0_*, *a_n_* and *b_n_* should be determined to minimise *E*. Therefore, differentiating from both sides of eq. (3) results the following set of equations:

| $dE= \frac{\partial E}{\partial a_{0}}da_{0}+\frac{\partial E}{\partial a_{n}}da_{n}+\frac{\partial E}{\partial b_{n}}db_{n}=0$ | (4) |
| --- | --- |
| $\frac{\partial E}{\partial a_{0}}= 0, \frac{\partial E}{\partial a_{n}}=0, \frac{\partial E}{\partial b_{n}}=0$ | (5) |
| $\frac{\partial E}{\partial a_{0}}= \sum_{i=1}^{m} \left[ y_{i}-(a_{0}+\sum_{n=1}^{N} (a_{n}cos\omega_{n}x_{i}+b_{n}sin\omega_{n}x_{i})) \right]=0$ | (6) |
| $\frac{\partial E}{\partial a_{n}}=\sum_{i=1}^{m} \left[ \left( y_{i}-\left( a_{0}+\sum_{n=1}^{N} (a_{n}cos\omega_{n}x_{i}+b_{n}sin\omega_{n}x_{i}) \right) \right)\left( \sum_{n=1}^{N} cos\omega_{n}x_{i} \right) \right]=0$ | (7) |
| $\frac{\partial E}{\partial b_{n}}=\sum_{i=1}^{m} \left[ \left( y_{i}-\left( a_{0}+\sum_{n=1}^{N} (a_{n}cos\omega_{n}x_{i}+b_{n}sin\omega_{n}x_{i}) \right) \right)\left( \sum_{n=1}^{N} sin\omega_{n}x_{i} \right) \right]=0$ | (8) |

Considering Eq. 5-8, a set of linear equations will be achieved as follows:

$\left[ \begin{matrix} \sum1 & \sum cos\omega_{1}x_{i} & \sum sin\omega_{1}x_{i} & . & . & . & \sum cos\omega_{n}x_{i} & \sum sin\omega_{n}x_{i} \\ \sum cos\omega_{1}x_{i} & \sum{cos}^{2}\omega_{1}x_{i} & \sum sin\omega_{1}x_{i}cos\omega_{1}x_{i} & . & . & . & \sum cos\omega_{n}x_{i}cos\omega_{1}x_{i} & \sum sin\omega_{n}x_{i}cos\omega_{1}x_{i} \\ \sum sin\omega_{1}x_{i} & \sum cos\omega_{1}x_{i}sin\omega_{1}x_{i} & \sum{sin}^{2}\omega_{1}x_{i} & . & . & . & \sum cos\omega_{n}x_{i}sin\omega_{1}x_{i} & \sum sin\omega_{n}x_{i}sin\omega_{1}x_{i} \\ . & . & . & . & . & . & . & . \\ . & . & . & . & . & . & . & . \\ . & . & . & . & . & . & . & . \\ \sum cos\omega_{n}x_{i} & \sum cos\omega_{1}x_{i}cos\omega_{n}x_{i} & \sum sin\omega_{1}x_{i}cos\omega_{n}x_{i} & . & . & . & \sum{cos}^{2}\omega_{n}x_{i} & \sum sin\omega_{n}x_{i}cos\omega_{n}x_{i} \\ \sum sin\omega_{n}x_{i} & \sum cos\omega_{1}x_{i}sin\omega_{n}x_{i} & \sum sin\omega_{1}x_{i}sin\omega_{n}x_{i} & . & . & . & \sum cos\omega_{n}x_{i}sin\omega_{n}x_{i} & \sum{sin}^{2}\omega_{n}x_{i} \end{matrix} \right]\left[ \begin{matrix} a_{0} \\ a_{1} \\ b_{1} \\ . \\ . \\ . \\ a_{n} \\ b_{n} \end{matrix} \right]=\left[ \begin{matrix} \sum y_{i} \\ \sum y_{i}cos\omega_{1}x_{i} \\ \sum y_{i}sin\omega_{1}x_{i} \\ . \\ . \\ . \\ \sum y_{i}cos\omega_{n}x_{i} \\ \sum y_{i}sin\omega_{n}x_{i} \end{matrix} \right]$

(9)

In the matrix above, the summation bound is *i=1,m*, in which m is the number of (*x*,*y*) data the curve-fitting is done on them. Writing the abovementioned set of equations in a concise format, the following form will be resulted:

| $FA=Y$ | (10) |
| --- | --- |

In which *F* is the matrix of coefficient, *A* is the matrix of unknown and *Y* is the matrix of constants. In order to find the matrix of unknown, which involves coefficients of Fourier series, the Gauss-Seidel method was employed.

# Three-element Windkessel Model

For the outlets three-element Windkessel model (RCR) was used to set a proper 0D-3D coupling. Eq. (11) defines the RCR model through a first order Ordinary Differential Equation (ODE) as described below.

| $\left( 1+\frac{R_{p}}{R_{d}} \right)Q\left( t \right)+R_{p}C\frac{dQ(t)}{dt}=\frac{P(t)}{R_{d}}+C\frac{dP(t)}{dt}$ | (11) |
| --- | --- |

| 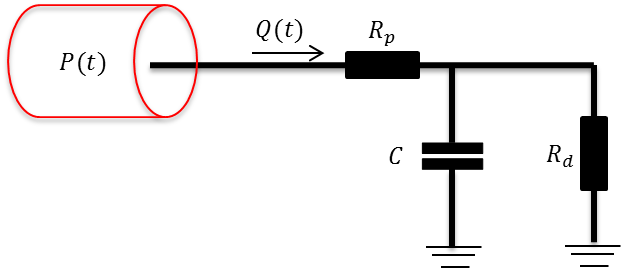 |
| --- |
| Figure 4. A schematic of three-element Windkessel model |

In Eq. (11), *R_p_* and *R_d_* are proximal and distal resistances, respectively, and *C* is the capacitance of each branch; furthermore, *P*(*t*) and *Q*(*t*) are the outlet pressure and flow rate, respectively. Table 1 shows all the values for the RCR circuit. It should be noted that RCR parameters were tuned for the volunteer, who participated in this study.

Eq. (11) was discretised implicitly, using first order backward Euler method as follows:

| $P^{n}=\frac{{(R}_{d}\Delta t+R_{p}\Delta t+CR_{p}R_{d})Q^{n}-CR_{p}R_{d}Q^{n-1}+CR_{d}P^{n-1}}{CR_{d}+\Delta t}$ | (12) |
| --- | --- |

In eq. (12), $\Delta t$ denotes timestep size, while n and n-1 superscripts define two consecutive time points at the current and previous moments, respectively. Thereafter, the discretised model was implemented by writing several User Defined Functions (UDF). The codes were scripted in FORTRAN programming language environment by employing relevant macros for CFX Expression Language (CEL). In particular, eight UDFs were written, one as the master code, which was controlling the seven other codes for each branch. The algorithm of the coding is shown below:

| 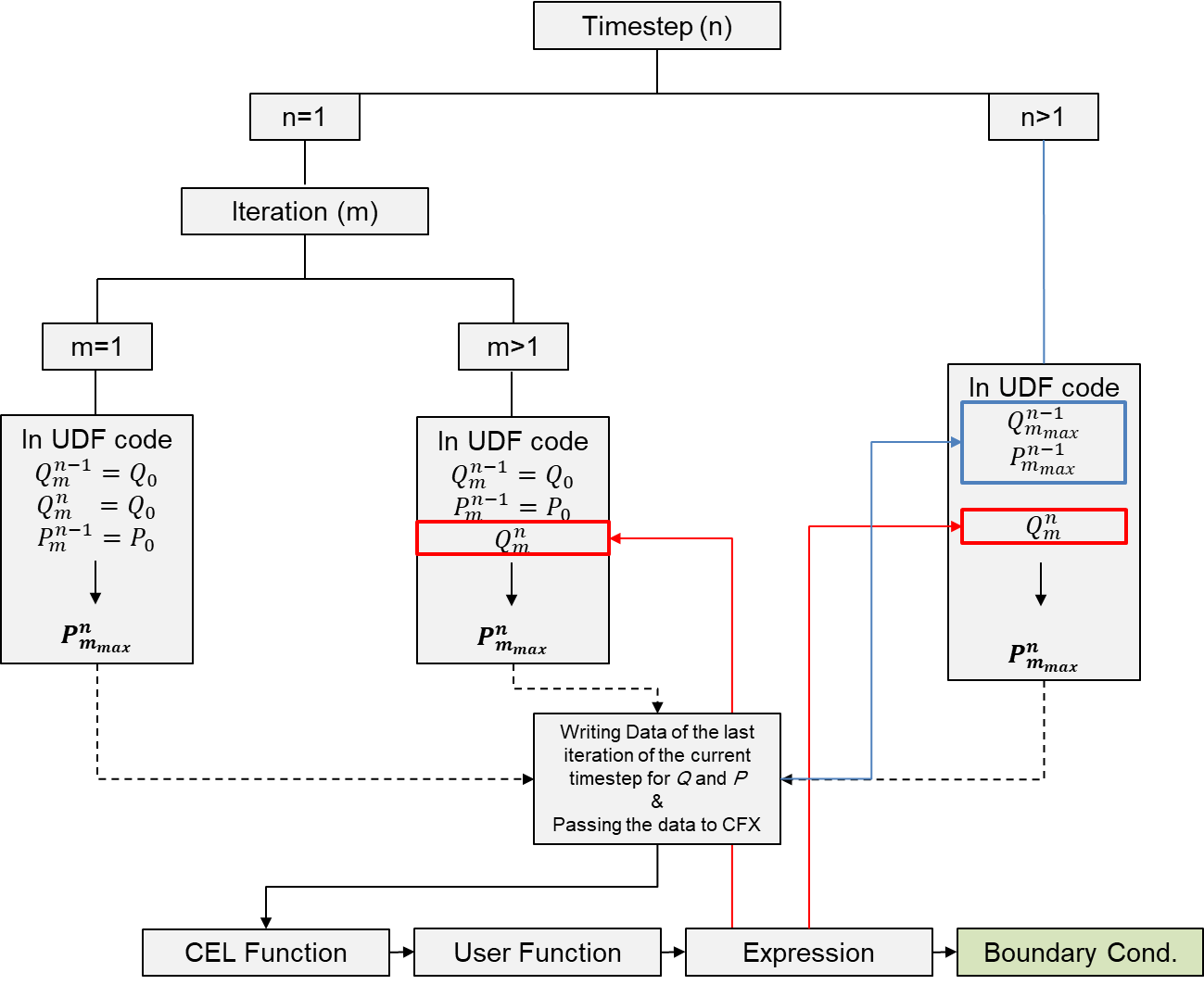 |
| --- |
| Figure 5. UDF algorithm coupled with ANSYS-CFX |

# Estimation of RCR Parameters for Three-element Windkessel Model

In this section a brief summary about the procedure employed to tune RCR variables is described. The accurate method for tuning the Windkessel parameters goes through a regression analysis which requires the availability of pressure waveforms at the outlet of different branches. Due to imaging constraints and lack of flow data at the supra-aortic branches, the RCR values were estimated through the following steps.

Initially the total resistance is defined, using the mean pressure and flow rate in a cardiac cycle:

| $R_{tot}= \frac{P_{avg}}{Q_{avg}}$ | (13) |
| --- | --- |
| $P_{avg}=\frac{P_{systole}+2P_{diastole}}{3}$ | (14) |
| $Q_{avg}=\frac{1}{T}\int_{0}^{T} Q\left( t \right)dt$ | (15) |

Thereafter, using the surface area of each branch, the resistance of each is estimated as:

| $R_{i}=\frac{\sum_{j=1}^{n=7} A_{j}}{A_{i}}R_{tot}$ | (16) |
| --- | --- |

The next step is to find the total capacitance. It has been proven that for the afterload condition, two-element Windkessel model predicts pressure properly ^3^, therefore using the two-element Windkessel model, during the diastole, the pressure can be estimated as:

| $P\left( t \right)=ke^{-\frac{t}{RC_{tot}}}$ | (17) |
| --- | --- |

Employing two time points, one just at the end of systole ($\sim\frac{1}{3}t_{cc}$) and another one at the end of diastole, just before the start of systole, the value of *k* and total capacitance are defined. Once the total capacitance is found, the capacitance of each branch can be estimated through:

| $C_{i}=\frac{A_{i}}{\sum_{j=1}^{n=7} A_{j}}C_{tot}$ | (18) |
| --- | --- |

Table 1 shows the estimated parameters:

| Table 1. Three-element Windkessel model parameters for each outlet | | | |
| --- | --- | --- | --- |
| Parameter | *R_p_*  (Pa.s/m^3^) | *R_d_*  (Pa.s/m^3^) | *C*  (m^3^/Pa) |
| RCA | 6.71×10^8^­_­_ | 6.78×10^9^ | 1.64×10^-10^ |
| LCA | 6.71×10^8^ | 6.78×10^9^ | 2.36×10^-10^ |
| RCCA | 9.1×10^7^ | 9.23×10^8^ | 1.47×10^-9^ |
| RSCA | 9.63×10^7^ | 9.74×10^8^ | 1.4×10^-9^ |
| LCCA | 1.5×10^8^ | 1.52×10^9^ | 9×10^-10^ |
| LSCA | 1.31×10^8^ | 1.33×10^9^ | 1.02×10^-9^ |
| TAo | 2.48×10^7^ | 2.51×10^8^ | 5.4×10^-9^ |

# Lumped Model of Aorta, Left Heart and Systemic Circulation

For the parametric study of AF effects, the following lumped model was employed ^4^, which considers the Left Atrium (LA), Mitral Valve (MV), Left Ventricle (LV) and Aortic Valve (AV) that were coupled with aorta and systemic circulation. To study different phases inside the LA, i.e. reservoir, conduit and booster pump, the LA compliance has been modified as a time-variant parameter. The circuit can produce corresponding inlet waveform for the Left Ventricular Outflow Tract (LVOT) as the left heart parameters change, so the resultant waveform can be applied as inlet boundary condition at aortic root to investigate flow distribution/perfusion during AF.

| 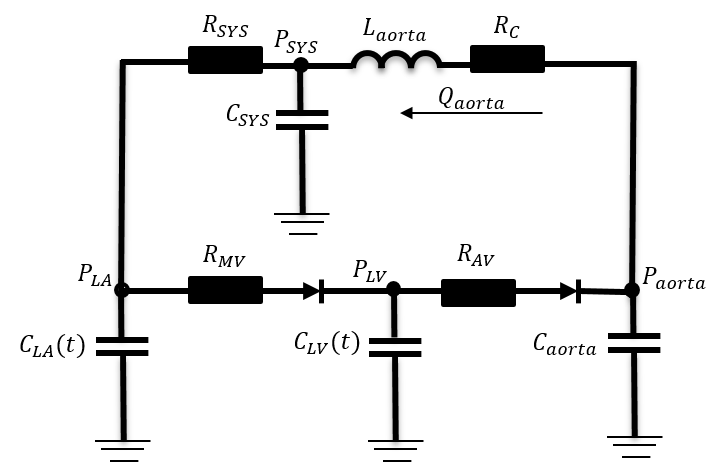 |
| --- |
| Figure 6. Lumped parameter model for the inlet |

Using this model, a set of first order ODEs were obtained, which can be arranged as follows:

| $\frac{d\boldsymbol{x}}{dt}=A\boldsymbol{x}+Bf$ | (19) |
| --- | --- |

In Eq. (19) ***x*** is the matrix of variable, *A* is the matrix of coefficient, *B* is the matrix of constants, while *f* is a ramp function that mimics the function of valves based on pressure difference across the MV and AV. All the matrices are defined below:

$$x=\left[ \begin{aligned} P_{LV}(t) \\ P_{LA}(t) \\ P_{SYS}(t) \\ P_{aorta}(t) \\ Q_{aorta}(t) \end{aligned} \right]$$

$$A=\left[ \begin{matrix} -\frac{\dot{C}_{LV}\left( t \right)}{C_{LV}\left( t \right)} & 0 & 0 & 0 & 0 \\ 0 & -\left( \frac{\dot{C}_{LA}\left( t \right)}{C_{LA}\left( t \right)}+\frac{1}{R_{SYS}C_{LA}} \right) & \frac{1}{R_{SYS}C_{LA}} & 0 & 0 \\ 0 & \frac{1}{R_{SYS}C_{SYS}} & -\frac{1}{R_{SYS}C_{SYS}} & 0 & \frac{1}{C_{SYS}} \\ 0 & 0 & 0 & 0 & -\frac{1}{C_{aorta}} \\ 0 & 0 & -\frac{1}{L_{aorta}} & \frac{1}{L_{aorta}} & -\frac{R_{aorta}}{L_{aorta}} \end{matrix} \right]$$

$$B=\left[ \begin{matrix} \frac{1}{C_{LV}\left( t \right)} & -\frac{1}{C_{LV}\left( t \right)} \\ -\frac{1}{C_{LA}\left( t \right)} & 0 \\ 0 & 0 \\ 0 & \frac{1}{C_{aorta}} \\ 0 & 0 \end{matrix} \right], f=\left[ \begin{matrix} \frac{1}{R_{MV}}F(P_{LA}(t)-P_{LV}(t)) \\ \frac{1}{R_{AV}}F(P_{LV}(t)-P_{aorta}(t)) \end{matrix} \right], F\left( y \right)=\left\{ \begin{matrix} y, if y>0 \\ 0, if y\leq0 \end{matrix} \right\}$$

In Eq. (19) and corresponding matrices, *P*(*t*) denotes the pressure, *Q*(*t*) is the flow rate, *R* refers to the resistance, and *C* is the compliance described as elastance function for the LV and LA, which correlates pressure and volume changes in an atrium and a ventricle as follows:

| $E_{LA}\left( t \right)=\frac{LAP(t)}{LAV\left( t \right)-V_{0}LA}=\frac{1}{C_{LA}(t)}$ | (20) |
| --- | --- |
| $E_{LV}\left( t \right)=\frac{LVP\left( t \right)}{LVV\left( t \right)-V_{0}LV}=\frac{1}{C_{LV}\left( t \right)}$ | (21) |

Where *E_LV_*(*t*) and *E_LA_*(*t*) are elastances of LV and LA, respectively; *LVP*(*t*) and *LAP*(*t*) are the LV and LA pressures, respectively, and *LVV*(*t*) and *LAV*(*t*) are associated to the volumes of LV and LA, while *V_0_LA* and *V_0_LV* are the theoretical volumes of LA and LV at zero pressure. The elastance of LA and LV can be estimated using proper mathematical descriptions. For the LV, the following equation is invoked:

| $E_{LV}\left( t \right)=\left( E_{LVmax}-E_{LVmin} \right)E_{n}\left( t_{n} \right)+E_{LVmin}$ | (22) |
| --- | --- |

In which $E_{n}\left( t_{n} \right)$ is the double-hill function, which initially proposed by Stergiopulos et al. ^5^ and is represented as follows:

| $E_{n}\left( t_{n} \right)=\alpha_{1}\left[ \frac{\left( \frac{t_{n}}{\alpha_{2}} \right)^{\beta_{1}}}{{1+\left( \frac{t_{n}}{\alpha_{2}} \right)}^{\beta_{1}}} \right]\left[ \frac{1}{1+\left( \frac{t_{n}}{\alpha_{3}} \right)^{\beta_{2}}} \right]$ | (23) |
| --- | --- |

In which $\alpha_{i}$ and $\beta_{j}$ are the model parameters that are defined as $\alpha_{1}=1.55, \alpha_{2}=0.7,\alpha_{3}=1.17, \beta_{1}=1.9 and \beta_{2}=21.9$. Moreover, $t_{n}=t/T_{max}$ in which $T_{max}=0.2+0.15t_{cc}$ and $t_{cc}$ is the interval of a cardiac cycle, which is taken to 0.8s for the normal heart function. $E_{LVmax}$ and $E_{LVmin}$ are maximal and minimal elastances associated to end-systolic and end-diastolic pressure volume relations as described in Eq. (20) and Eq. (21). For the LA elastance, the following equation is invoked:

| $E_{LA}\left( t \right)=\frac{\left( E_{LAmax}-E_{LAmin} \right)}{2}e_{LA}\left( t \right)+E_{LAmin}$ | (24) |
| --- | --- |

Where $e_{LA}\left( t \right)$ is described as follows ^2^:

| $e_{LA}\left( t \right)=\left\{ \begin{matrix} 0 & if 0\leq t\leq T_{ac} \\ 1-\cos\left( \frac{t-T_{ac}}{t_{cc}-T_{ac}}2\pi\right) & if T_{ac}<t_{cc} \end{matrix}, T_{ac}=0.80t_{cc} \right.$ | (25) |
| --- | --- |

| Table 2. Baseline values of the lumped model for the inlet | | |
| --- | --- | --- |
| Parameter | Values | Definition |
| Resistance (mmHg.s/ml) | | |
| ^a^*R_MV_* | 0.020 | Mitral valve resistance |
| ^b^*R_AV_* | 0.001 | Aortic valve resistance |
| ^c^*R_C_* | 0.100 | Characteristic resistance |
| ^b^*R_SYS_* | 1.0000 | Systemic vascular resistance |
| Inertance (mmHg.s^2^/ml) | | |
| ^b^*L_aorta_* | 0.0005 | Blood inertial effect inside the aorta |
| Compliance (ml/mmHg) | | |
| *C_LA_*(*t*) | 1/*E_LA_*(*t*) | Left atrium compliance |
| *C_LV_*(*t*) | 1/*E_LV_*(*t*) | Left ventricle compliance |
| ^c^*C_aorta_* | 0.1000 | Aortic compliance |
| ^c^*C_SYS_* | 1.5000 | Systemic compliance |
| Elastance (mmHg/ml) | | |
| ^b^*E_LVmin_* | 0.0500 | Minimum elastance of LV |
| ^b^*E_LVmax_* | 2.0000 | Maximum elastance of LV |
| ^a^*E_LAmin_* | 0.2000 | Minimum elastance of LA |
| ^a^*E_LAmax_* | 0.3000 | Maximum elastance of LA |
| Volume (ml) |  |  |
| ^b^*V_0_LV* | 10.0000 | Theoretical volume of LV at zero pressure |
| ^b^*V_0_LA* | 4.0000 | Theoretical volume of LA at zero pressure |
| ^a^Scarsoglio et al. (2014)^2^  ^b^Simaan et al. (2009)^4^  ^c^Tuned for this study, but in the range used by Simaan et al. (2009)^4^. | | |

In this study four AF-associated defects were examined. To reflect the effect of each one, several parameters were varied. In Table 3, the chosen values for each parametric study have been shown. Furthermore, the logic behind each one along with its relevant reference is provided in the main text.

| Table 3. Variable quantities for each AF-associated defect | | | | | |
| --- | --- | --- | --- | --- | --- |
| Case # | ELA_min_ | ELA_max_ | ELV_min_ | ELV_max_ | t_cc_ |
| Lack of AK | | | | | |
| ELAC1 | **0.002** | **0.002** | 0.05 | 2 | 0.8 |
| ELAC2 | **0.02** | **0.02** | 0.05 | 2 | 0.8 |
| ELAC3 | **0.2** | **0.2** | 0.05 | 2 | 0.8 |
| ELAC4 | **2** | **2** | 0.05 | 2 | 0.8 |
| ELAC5 | **20** | **20** | 0.05 | 2 | 0.8 |
| ELAC6 | **200** | **200** | 0.05 | 2 | 0.8 |
| LAR | | | | | |
| ELA1 | **0.002** | **0.003** | 0.05 | 2 | 0.8 |
| ELA2 | **0.02** | **0.03** | 0.05 | 2 | 0.8 |
| ELA3 | **0.2** | **0.3** | 0.05 | 2 | 0.8 |
| ELA4 | **2** | **3** | 0.05 | 2 | 0.8 |
| ELA5 | **20** | **30** | 0.05 | 2 | 0.8 |
| ELA6 | **200** | **300** | 0.05 | 2 | 0.8 |
| LVSD | | | | | |
| ELV1 | 0.2 | 0.3 | 0.05 | **0.3** | 0.8 |
| ELV2 | 0.2 | 0.3 | 0.05 | **0.5** | 0.8 |
| ELV3 | 0.2 | 0.3 | 0.05 | **1** | 0.8 |
| ELV4 | 0.2 | 0.3 | 0.05 | **1.5** | 0.8 |
| ELV5 | 0.2 | 0.3 | 0.05 | **2** | 0.8 |
| HFF | | | | | |
| 70 bpm | 0.2 | 0.3 | 0.05 | 2 | **0.8** |
| 100 bpm | 0.2 | 0.3 | 0.05 | 2 | **0.6** |
| 150 bpm | 0.2 | 0.3 | 0.05 | 2 | **0.4** |

# Cardiac Metrics

In the present study, three main cardiac metrics were employed that are introduced as follows:

Stroke Volume (SV) = End Diastolic Volume (EDV) – End Systolic Volume (ESV)

Ejection Fraction (EF) = (SV/EDV)_­_×100

Cardiac Output (CO) = SV×HR

# A Comparison of LV Function During AF with In-vivo Measurement

In Figure 7 a comparison is made between the current study and an in-vivo measurement^1^. The comparison displays the correlation between EF and ESP/SV, and between EF and ESP/ESV.

|  |  |
| --- | --- |
| (a) | |
| 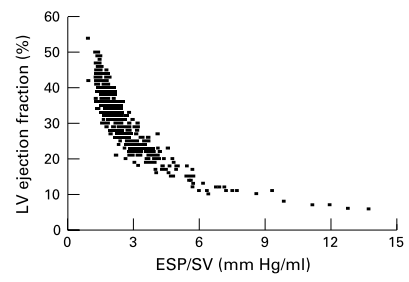 | 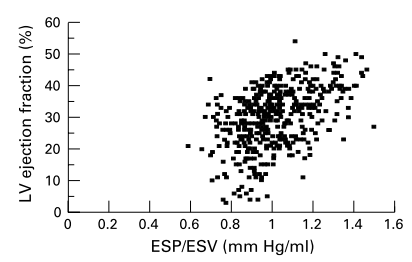 |
| (b) | |

Figure 7. Variations of EF with respect to End Systolic Pressure (ESP) to SV (ESP/SV), and ESP to End Systolic Volume (ESV) (ESP/ESV); (a) present study, (b) study by Muntinga et al.^1^ (figures are taken from the work by Muntinga et al.^1^).

# Comparison of Haemodynamic Parameters

As a complementary source of Endothelial Cell Activation Potential (ECAP) results in Figure 6 in the main body of the paper, Oscillatory Shear Index (OSI) and Time-averaged Wall Shear Stress (TAWSS) are presented in Figure 7 to compare impact of each through different AF abnormalities.

| Normal | 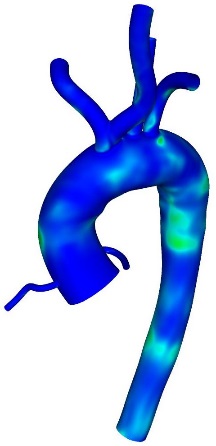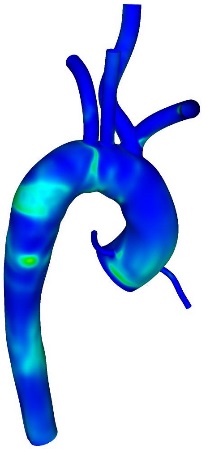 | 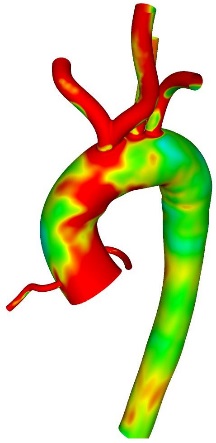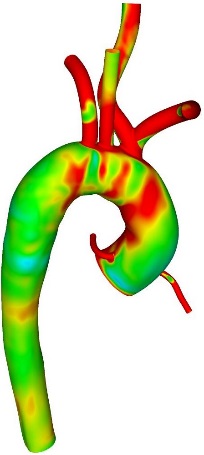 | 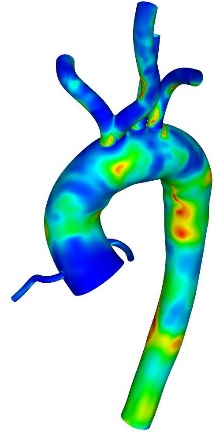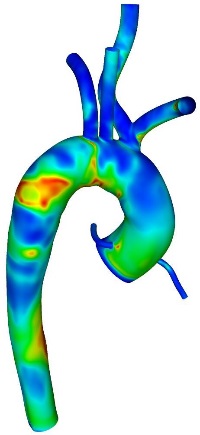 |
| --- | --- | --- | --- |
| ELA1 | 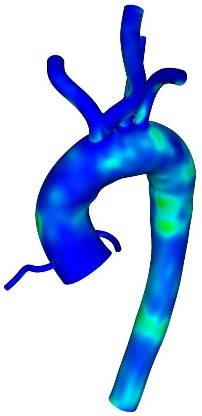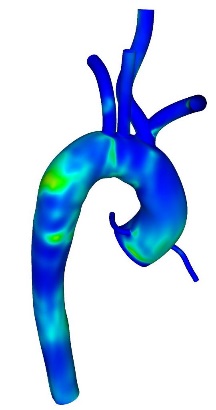 | 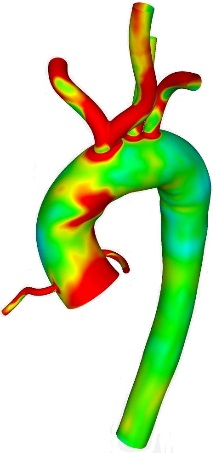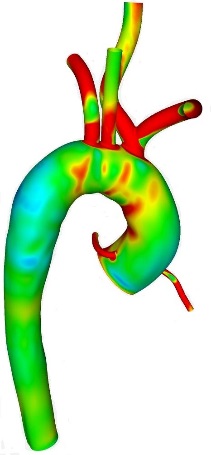 | 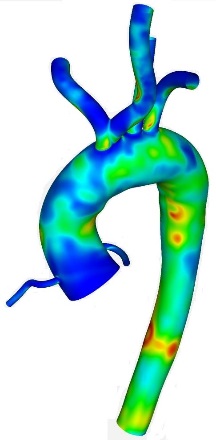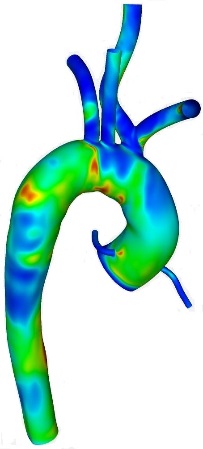 |
| ELA6 | 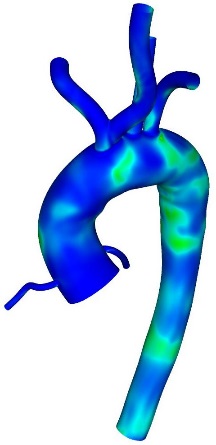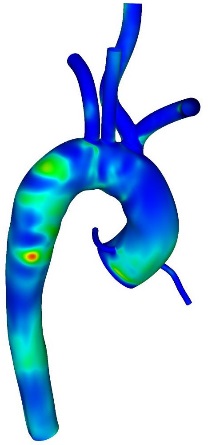 | 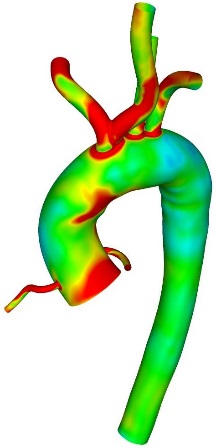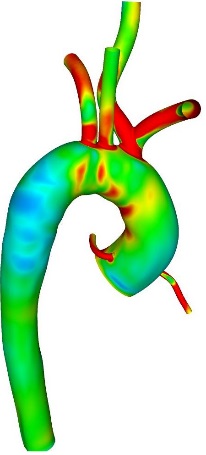 | 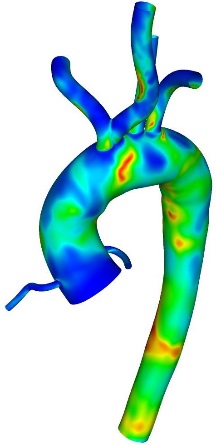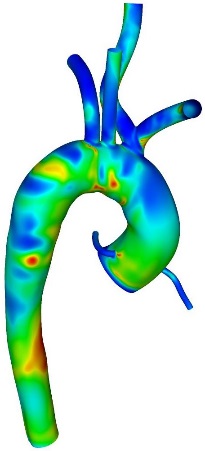 |
| ELV1 | 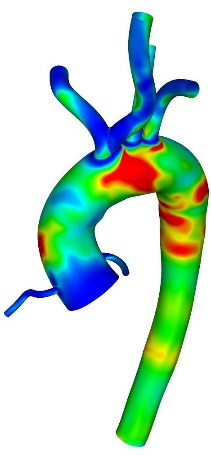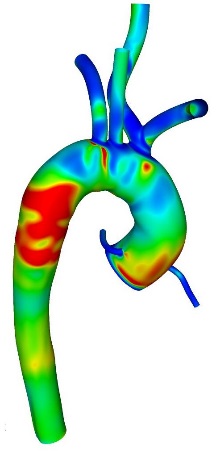 | 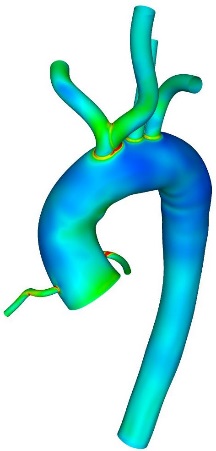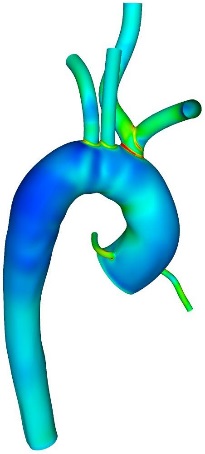 | 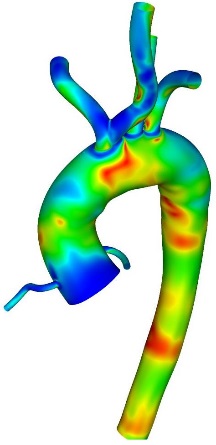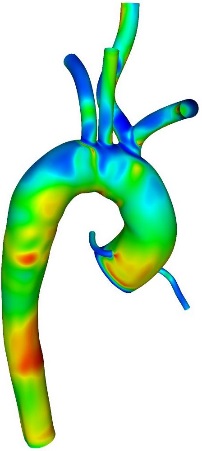 |
| ELV3 | 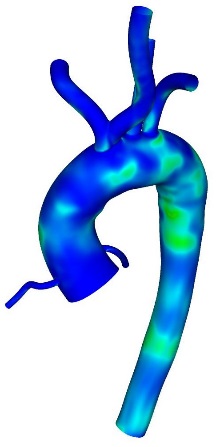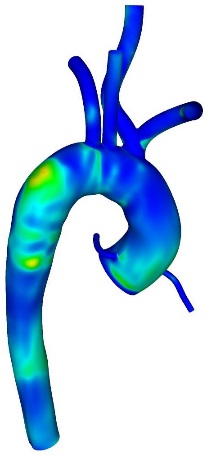 | 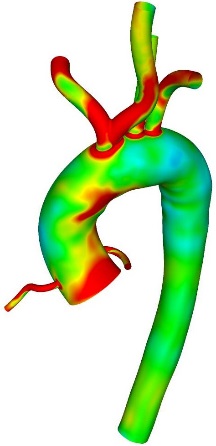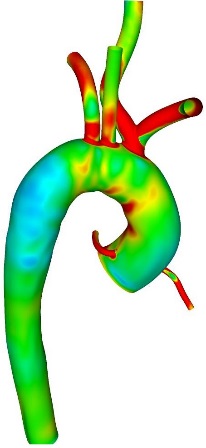 | 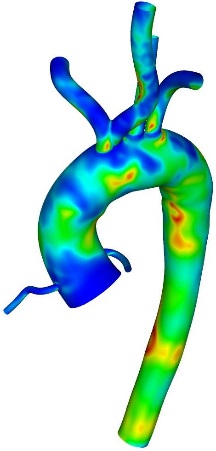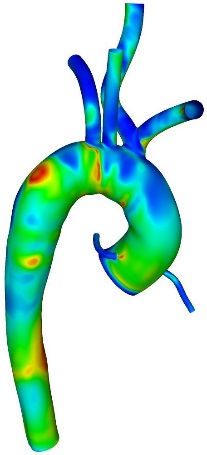 |
| 100bpm | 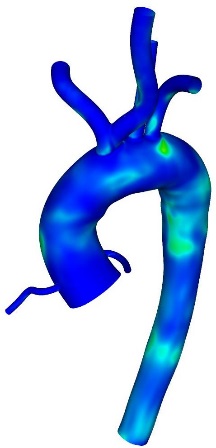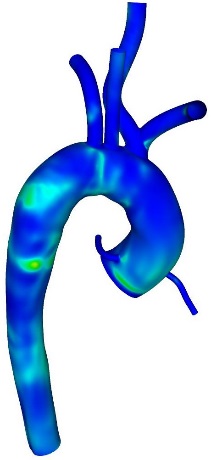 | 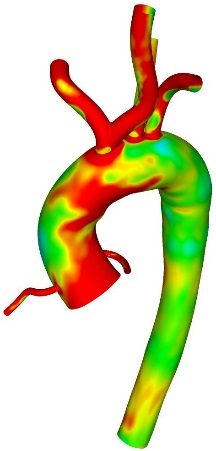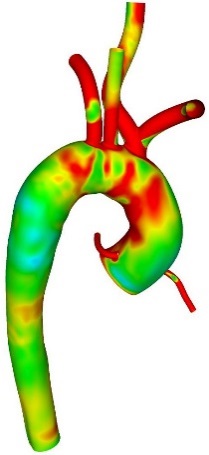 | 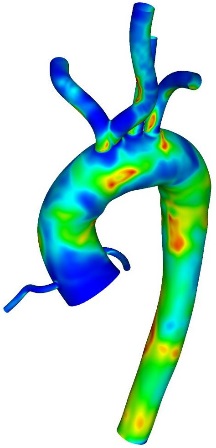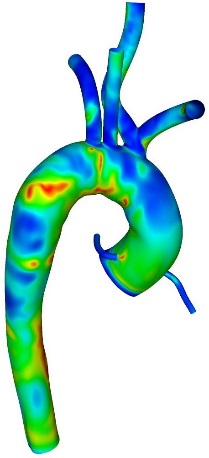 |
| 150bpm | 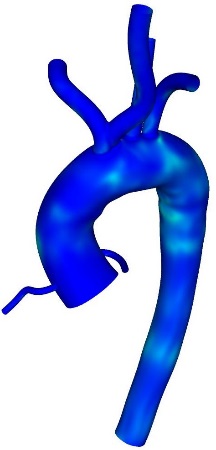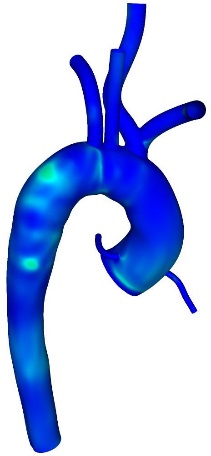 | 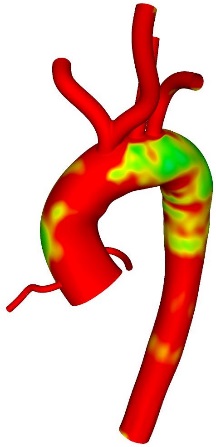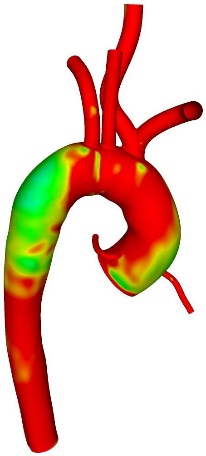 | 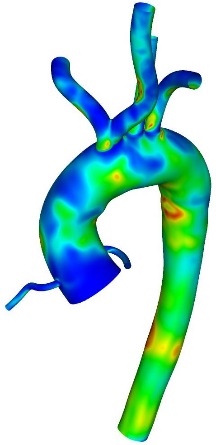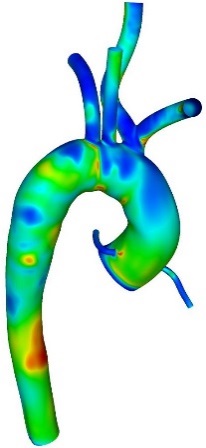 |
|  | 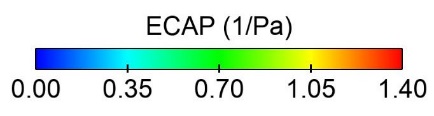 | 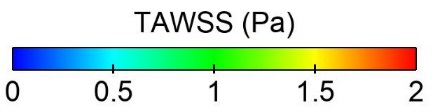 | 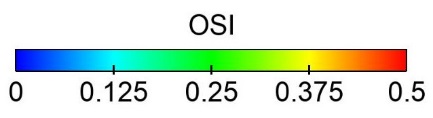 |

Figure 8. Comparison of ECAP, TAWSS and OSI during four AF-associated defects including left atrial remodelling, left ventricular systolic dysfunction and high frequency fibrillation.

**References**

1. Muntinga, H. J., A. T. M. Gosselink, P. K. Blanksma, P. J. De Kam, E. E. Van Der Wall, and H. J. G. M. Crijns. Left ventricular beat to beat performance in atrial fibrillation: Dependence on contractility, preload, and afterload. *Heart* 82:575–580, 1999.

2. Scarsoglio, S., A. Guala, C. Camporeale, and L. Ridolfi. Impact of atrial fibrillation on the cardiovascular system through a lumped-parameter approach. *Med. Biol. Eng. Comput.* 52:905–920, 2014.

3. Shi, Y., P. Lawford, and R. Hose. Review of Zero-D and 1-D Models of Blood Flow in the Cardiovascular System. *Biomed. Eng. Online* 10:33, 2011.

4. Simaan, M. A., A. Ferreira, S. Chen, J. F. Antaki, and D. G. Galati. A dynamical state space representation and performance analysis of a feedback-controlled rotary left ventricular assist device. *IEEE Trans. Control Syst. Technol.* 17:15–28, 2009.

5. Stergiopulos, N., J. J. Meister, and N. Westerhof. Determinants of stroke volume and systolic and diastolic aortic pressure. *Am. J. Physiol.* 270:H2050-9, 1996.
